# Phylogenetic niche conservatism explains an inverse latitudinal diversity gradient in freshwater arthropods

**DOI:** 10.1038/srep26340

**Published:** 2016-05-24

**Authors:** Jérôme Morinière, Matthew H. Van Dam, Oliver Hawlitschek, Johannes Bergsten, Mariano C. Michat, Lars Hendrich, Ignacio Ribera, Emmanuel F.A. Toussaint, Michael Balke

**Affiliations:** 1 SNSB-Bavarian State Collection of Zoology, Münchhausenstrasse 21, 81247, Munich, Germany; 2 Institute of Evolutionary Biology (CSIC-Universitat Pompeu Fabra), Passeig Maritim de la Barceloneta 37, 08003 Barcelona, Spain; 3 Department of Zoology, Swedish Museum of Natural History, Box 50007, SE-10405, Stockholm, Sweden; 4 IBBEA, CONICET-UBA, Laboratory of Entomology-DBBE_FCEN, University of Buenos Aires, Buenos Aires, Argentina; 5 Department of Ecology & Evolutionary Biology & Division of Entomology, Biodiversity Institute, University of Kansas, Lawrence, KS 66045, USA; 6 GeoBioCenter, Ludwig-Maximilians-Universität München, Munich, Germany

## Abstract

The underlying mechanisms responsible for the general increase in species richness from temperate regions to the tropics remain equivocal. Many hypotheses have been proposed to explain this astonishing pattern but additional empirical studies are needed to shed light on the drivers at work. Here we reconstruct the evolutionary history of the cosmopolitan diving beetle subfamily Colymbetinae, the majority of which are found in the Northern hemisphere, hence exhibiting an inversed latitudinal diversity gradient. We reconstructed a dated phylogeny using 12 genes, to investigate the biogeographical history and diversification dynamics in the Colymbetinae. We aimed to identify the role that phylogenetic niche conservatism plays in the inversed diversification pattern seen in this group. Our results suggest that Colymbetinae originated in temperate climates, which supports the hypothesis that their distribution is the result of an ancestral adaptation to temperate environmental conditions rather than tropical origins, and that temperate niche conservatism can generate and/or maintain inverse latitudinal diversity gradients.

The increase in species diversity with decreasing latitude, or high tropical species richness, is an ecological pattern that has long intrigued naturalists^1^. Best known as the latitudinal diversity gradient (LDG), numerous empirical studies have investigated the relative contribution of macroevolutionary drivers such as diversification rate dynamics and biogeographical history^2^,^3^,^4^,^5^,^6^,^7^,^8^. It was suggested, for example, that LDGs resulted from a reduced number of lineages evolving adaptations to cold and dry climates with strong seasonal oscillations typical of non-tropical areas^9^. Tropical regions are also viewed as both cradles of diversity that continuously generate new species, and museums that harbour ancient species persisting throughout geological times^10^,^11^. Several hypotheses attempted to summarize the available evidence explaining the origin and evolution of the LDG^11^,^12^,^13^,^14^,^15^. The ‘out of the tropics’ (OTT) hypothesis suggests that tropical regions harbour a high number of species eventually expanding their distributional ranges towards the poles and/or high altitudes^13^,^14^. However, according to the tropical niche conservatism (TNC^16^,^17^) and the tropical conservation hypothesis (TCH - Zanne *et al.* 2014^18^), dispersal out of the tropics towards temperate zones is limited by the tendency of lineages to retain their tropical niche-related traits throughout the speciation process. These hypotheses both focus on the historical biogeography and/or physiological niche conservatism limiting the expansion of clades out of the tropics.

In contrast to niche conservatism, other hypotheses are based on the assumption that tropical environments are geologically older, have occupied larger areas and have been more stable over time (as summarized in^18^). The time for speciation hypothesis (TFS) predicts that the time required for speciation to build up diversity in a region explains the high species diversity in the tropics in contrast to the geologically younger and less stable temperate environments^19^. The Centre of Origin (COO) hypothesis suggests that lineage diversification took place in older and historically larger tropical regions, resulting in higher species richness compared to the younger temperate regions^20^. Other hypotheses invoke higher rates of tropical speciation *versus* higher rates of temperate extinction^21^. Recent studies suggest that major environmental change (e.g. glaciations or volcanism) might have supported LDGs^2^,^22^. Instead of focussing on niche conservatism, these hypotheses aim at explaining LDGs as results of migration, speciation and extinction rates. While these hypotheses generally focus on explaining high tropical biodiversity, similar mechanisms might also serve as explanations as to why some taxa are more diverse in temperate regions^18^.

These hypotheses are not necessarily mutually exclusive, but rather provide a framework for a comprehensive explanation of complex and partly idiosyncratic processes^15^. For instance, Wiens *et al.*^23^ suggest that the higher tropical diversity of Old World Ranidae frogs can neither be accounted for by accelerated tropical speciation nor by higher temperate extinction, but rather by relatively recent colonization of the temperate regions. This highlights the importance of comprehensive biogeographical analyses in a phylogenetic context, which have been conducted in many case studies on LDGs.

However, few comprehensive studies have investigated the opposite case, namely the inverse Latitudinal Diversity Gradient (iLDG). Taxa presenting an iLDG exhibit relatively high species richness in temperate areas compared to the tropics. This pattern was first shown for a number of Holarctic bird families^24^, later for parasitic ichneumonid wasps^25^,^26^,^27^,^28^, marine benthic algae^29^, aphids^30^, Emydidae turtles^19^, shallow-water molluscs (as summarized in^31^), Holarctic tree frogs^32^, New World Lampropeltini snakes^18^, and cosmopolitan Poaceae grasses^9^. The macroevolutionary and ecological factors during lineage diversification that led to iLDGs remain little explored to date. Thus, there is a need for additional empirical studies based on comprehensive, large scale datasets to study the triggers of iLDG^18^,^31^. Molecular phylogenetics and recent methodological developments provide tools for a more accurate inference of diversification dynamics and biogeographical histories. These can then be used to empirically test the origins and causes of heterogeneous clade diversity distributions^2^,^23^.

Here, we study Colymbetinae diving beetles, which are comprised of 140 described species occurring in a wide variety of aquatic habitats on all continents but Antarctica. Colymbetinae show a marked iLDG, being most diverse in the Holarctic region with decreasing species diversity towards the Equator, but with a conspicuous equatorial “peak” in the Eastern Old World tropics (Figure 1). They are almost completely absent from tropical lowlands but occur in tropical montane and alpine habitats, and in the southern temperate regions^33^,^34^,^35^.

We aim to (*i*) infer the temporal and spatial origins of the group using fossil-based calibration and likelihood-based historical biogeography methods, (*ii*) test for possible diversification rate shifts as an explanation for the iLDG, (*iii*) calculate the extent of phylogenetic niche conservatism within the lineage and (*iv*) identify the putative mechanisms explaining the iLDG as well as species ecological preferences during lineage diversification.

## Material and Methods

### Taxon sampling

We used 87 Colymbetinae species (Table S1) mostly preserved in 96% ethanol, representing 62% of the ca. 140 described species and all extant genera and subgenera^36^. We also included 17 species of Agabinae, the sister-group of Colymbetinae^37^ as outgroups. We rooted the tree with *Batrachomatus daemeli* (Sharp 1882) (Matinae), a related subfamily clearly outside Colymbetinae plus Agabinae.

### Molecular biology

Genomic DNA was extracted and purified using the Qiagen DNeasy tissue kit (Qiagen, Hilden, Germany). DNA samples were then used to amplify five mitochondrial gene fragments: *12S* (352bp in the combined dataset), *16S* (797bp), *cytochrome b* (326bp), *cytochrome oxidase 1* (1,336bp) and *cytochrome oxidase 2* (527bp)) in addition to seven nuclear gene fragments: *18S* (1,951bp), *arginine kinase* (674bp), *enolase* (677bp), *wingless* (496bp), *elongation factor 1 alpha* (553bp), *histone 4* (159bp) and *carbamoyl-phosphate synthetase 2* (815bp) using standard procedures following Balke *et al.*^33^ and Tänzler *et al.*^38^. PCR products were purified and processed for sequencing, using BigDye v3.1 (ABI, Darmstadt, Germany). Assembling and editing of the sequences were performed using Sequencher 4.10.1 (Gene Codes, Ann Arbor, MI, USA). All protein coding genes were then imported into Mesquite v2.75 (Maddison and Maddison 2015^39^) in order to translate to amino acids and screen for anomalies. Gene alignments were concatenated using Geneious R8 (Biomatters, http://www.geneious.com). All sequences are available online (BOLD Process IDs ZSMDB032-15 - ZSMDB138-16, Genbank Accession Numbers KJ638060 - 607997).

### Phylogenetic inference

Phylogenetic inferences were conducted using maximum parsimony (MP), maximum likelihood (ML) and Bayesian inference (BI). The MP analyses were carried out using the TNT v1.1 program^40^ applying the *Tree Ratchet*, *Tree Fusing* and *Tree Drifting* Sectorial Searches and 1000 Jackknife replicates (P = 36) to assess the stability of nodes. ML analyses were conducted using RAxMLGUI v.0.93^41^ with the *autoFC* command for automatic determination of sufficient fast bootstrap repeats. A partitioning scheme with protein coding genes partitioned into 1^st^, 2^nd^, and 3^rd^ codon positions, as well as corresponding models of substitutions were obtained using PartitionFinder v1.1.1^42^ (Table S2). BI analyses of the combined dataset were performed on the workstations and the cluster of the entomology department of the Bavarian State Collection of Zoology with MrBayes v.3.2^43^. The analyses started with a random tree and consisted of two runs each with four chains (Markov Chain Monte Carlo, one cold and three incrementally heated) running for 50 million generations and sampling every 1000 cycles. A conservative burn-in of 25% was applied after checking that convergence was reached through the split frequencies of each run in Tracer 1.5^44^ (Table S3). The resulting trees were then combined to calculate a 50% majority rule consensus topology mapping the posterior probabilities (PP) of each node.

### Estimation of divergence times

We estimated absolute divergence times using a combination of calibration sets in order to improve the robustness of the estimates. At first, we applied three substitution rates calculated for the 3’ end of *COI* gene in recent studies focussing on Coleoptera lineages (as in ref. 45). We used an interval comprising these three distinct rate values, instead of performing multiple independent analyses applying each rate individually (recent examples are^38^,^45^). We applied the mean substitution rate from a dated phylogeny of carabid beetles (r = 0.0145; r is the substitutions per site per million years per lineage, subs/s/Myr/l), based on multiple geological and fossil records. We then used the divergence rate calculated for Tenebrionidae beetles (r = 0.0177 subs/s/Myr/l). Finally, we used the rate calculated for the *Rhantus suturalis* clade (r = 0.0195 subs/s/Myr/l). We applied the introduced interval (0.0145–0.0195 subs/s/Myr/l) to specify a normal and a uniform distribution on the *ucld.mean* in BEAUTi v1.5.4^44^. By incorporating the means of the three rates, we took into account the substitution rate heterogeneity across beetle lineages. For the very same analysis, three different fossil calibrations were implemented in BEAUTi. To constrain the root of the tree, we applied a uniform distribution (Lower = 0, Upper = 155), restricting the root not to be older than 155 million years ago (Ma). This is the approximate age of the oldest known dytiscid fossil †*Palaeodytes guttata*. We used a uniform distribution for two fossils, namely †*Colymbetes aemulus* Heer from the Miocene and †*Agabus rathbuni* Scudder from the Oligocene. The two fossils were respectively used to enforce a minimum constraint on the crown groups of the genus *Colymbetes* and the subfamily Agabinae. The youngest estimate of the geological strata they were embedded in was used as a minimum bound for each calibration (11.6 for †*Colymbetes aemulus* and 37.2 for †*Agabus rathbuni*), whereas the maximum bounds were set by the age of the defined root (155). The *Tree Model* was set to a birth-death model in an analysis consisting of 50 million generations sampled every 5000 generations. BEAST v.1.7^46^ analyses were conducted on the workstations and the cluster of the entomology department of the Bavarian State Collection of Zoology. As a starting tree for the BEAST analysis, the BI topology was used in order to optimize the search of optimal ages by starting at high likelihood in topology space. The convergence of the runs was investigated using statistics in Tracer inclusive ESS values. A conservative burn-in of 25% was applied after checking the log-likelihood curves and a maximum credibility tree with median ages and their 95% highest posterior density (HPD) were subsequently generated using TreeAnnotator 1.7.4^46^.

### Hypothesis testing overview

In order to test the hypothesis of iLDGs being a result of temperate niche conservatism, we needed to test for congruence between the biogeographical origins and the historical climatic preferences for Colymbetinae. To do this, we gathered information on their modern distributions and performed historical biogeographical analyses to test for their ancestral ranges. To test for Phylogenetic Niche Conservatism (PNC), we performed ancestral state reconstructions for temperature and precipitation. We then used the biogeographical results in combination with the ancestral climate reconstructions to ultimately test for evidence of PNC triggering the iLDG.

### Historical biogeography analyses

We used the R package *BioGeoBEARS* to test between different biogeographical model-based approaches^47^,^48^. As *BioGeoBEARS* requires an ultrametric tree, we used the BEAST chronogram from which all outgroup species (including Agabinae) were pruned using Mesquite v.2.75. Information on their current distributions was taken from the world catalogue of Dytiscidae^36^. Seven biogeographical regions were defined, namely Nearctic (A), Neotropics (B), Western Palaearctic (C), Afrotropics (D), Eastern Palaearctic (West of the Ural mountains including Asia and the Oriental region) (E), Australia (F) and the Pacific region (G). In an effort to reduce the large amount of computation time, the Eastern Palaearctic was merged with the South Asian region, as only a few species inhabit the latter area. We compared three main models: the Dispersal-Extinction-Cladogenesis model (DEC^49^,^50^), the DIVA-like model^51^ and the BAYAREA-like model^52^ to infer the ancestral ranges and colonization history of the Colymbetinae. They were implemented with and without the jump dispersal parameter (J), where ranges can change to include new areas during speciation^48^, for a total of six distinct models (DEC, DEC + J, DIVA-like, DIVA-like + J, BAYAREA-like, BAYAREA-like + J). We first ran a model in which dispersal between regions was not penalized (all rates are 1.0) and later designed and ran other models with varying dispersal probabilities reflecting past climatic and geological events^53^,^54^ . These included (i) adjacency matrix constraints, (ii) varying dispersal probabilities over time and (iii) a combination of both. After comparing models using AICc weights, we ran additional tests to explore what effect varying stringency on long-distance dispersal (LDD) had on model selection. In the second set of analyses, the LDD events were penalized differently in each model, in particular for LDD events between the Neotropical and Afrotropical or Pacific regions, as well as dispersal between the Palaearctic and the Nearctic regions (Table 1). We chose four time slices (0–5, 5–30, 30–45 and 45–70 MYA) for all models to account for major climatic and geological events throughout the entire evolution of the group. To reduce the set of possible regions, the maximum number of ancestral regions for each node was set to four.

### Climate Niche Modelling

Distribution data of the species used was obtained from the entomological collection of the Bavarian State Collection of Zoology (ZSM), the Global Biodiversity Information Facility (GBIF – www.gbif.de) and the catalogue of Dytiscidae^36^. Georeferenced coordinates were obtained using www.gpso.de/maps. The number of occurrence points ranged from 10 to 127. For species with very restricted ranges (endemic to small islands or isolated mountain tops) 10 points were evenly scattered across the spatial extents of these geographic features (see examples of the same procedure in refs 55 or 56). Current climatic conditions data (~1950–2000) on 19 BioClim variables was downloaded at a resolution of 30 arc-seconds (ca. 1km) (http://www.worldclim.org). We used a subset of the BioClim variables to capture information about general attributes of the climate that are known to be relevant for dytiscids (temperature and precipitation) (Table 2)^57^,^58^.

We used *Maxent* v.3.3.3k^59^,^60^ to predict habitat suitability given our environmental variables and georeferenced records. *Maxent* has previously been shown to work well with small number of occurrence points (minimum 10)^55^,^56^,^61^. We used the default settings for model training in *Maxent*. We evaluated model performance using AUC (Area Under the Receiver Operator Curve). For AUC model evaluation we withheld 20% of the samples for testing. AUC values range from 0.5, which are no better than a random coin flip, to 1.0 for optimal predictive accuracy of presence versus absence. We used the R packages *raster*, *maps*, *rgdal*, *maptools*, *sp* and *dismo* for this process^62^. The environmental niche modelling showed that BIO1, BIO2 and BIO4 contributed the most to the model predictions among the different biogeographical regions (Figure S1). The results of the niche models for each species can be found in the supplementary information. The calculated AUC values ranged from 0.95 to 0.53, 90% of the species had values > 0.5 (Table S4). These niche models allowed us to obtain an estimate of suitable environmental conditions for each species.

### Predicted Niche Occupancy (PNO) and Niche overlap

To identify whether clades can rapidly change their environmental preferences, we calculated the mean for each species from the range of values in our predicted niche occupancy (PNO) profiles. The PNO takes the likelihood surface from the *Maxent* output and relates it to the raster input layers to calculate a species probability of occurrence at a given environmental value. We cropped the BioClim layers and models produced by *Maxent* according to each species’ spatial extent in order to accommodate limited computational memory. PNO profiles were first constructed in the R package *phyloclim*^63^. One hundred random samples were then drawn from the PNO profile and then the mean was calculated as in Evans *et al.*^56^. The mean calculated from the PNOs was used in further ancestral state reconstructions. PNOs for each BioClim variable used were then merged and binned by 10 (1°C/10mm). Niche overlap was computed in *phyloclim* from the PNO profiles, using the summary statistics Schoener’s D and Hellinger Distances as in Warren *et al.*^64^. As we were interested in looking at the niche overlap between biogeographical regions, species were combined according to their geographic region and a mean PNO was calculated, which gave an estimate of the niche overlap between regions.

### Testing for Phylogenetic Niche Conservatism

In order to test for PNC, we calculated the environmental mean for the given BioClim variables for each species in *phyloclim*. We tested for PNC using recently proposed methods^65^,^66^. In order to test for evidence of a phylogenetic signal in the bioclimatic variables used here, we first calculated Blomberg’s K values^67^. We then tested for PNC amongst 3 evolutionary models of trait evolution: Brownian Motion (BM; genetic drift), White Noise (WN; no phylogenetic signal) and Ornstein-Uhlenbeck (OU; stabilizing selection)^68^. Support of the WN model indicates that traits are evolving independently without phylogenetic signal, whereas the BM and OU models indicate that traits evolve with an underlying phylogenetic pattern. Selection of the OU over the BM model indicates more stabilizing selection over drift.

### Diversification rate analyses

To investigate potential diversification rate shifts in the evolution of Colymbetinae while taking into account the missing taxon sampling in our phylogenetic reconstruction we used the function ‘*bd.shifts.optim’* in the R package *TreePar* (as in ref. 45). We used the BEAST chronogram as input and fitted several birth-death models including 0 (null model, constant-rate model) to multiple diversification rate shifts during the evolution of the group. We then tested different models ranging from 0 to 5 rate shifts. All analyses were carried out with the following non-default settings: taxon sampling 87/140, start = 0, end = 56.0 and grid = 0.1 Myr for a fine-scale estimation of rate shifts. We finally calculated AICc scores and computed Likelihood Ratio Tests (LRT) to select the best-fit between the different models allowing incrementally more shifts during the evolution of the clade.

We also used Bayesian Analysis of Macroevolutionary Mixtures (BAMM^71^) and its R implementation *BAMMtools*^72^ to identify clades with higher or lower speciation rates in the Colymbetinae phylogeny. We performed multiple BAMM runs on the BEAST chronogram, with five million generations of Markov Chain Monte Carlo (MCMC) sampling per run and sampling evolutionary parameters every 1000 generations. We assessed the convergence of the different BAMM runs by computing effective sample sizes of log-likelihoods, number of processes and evolutionary rate parameters using the package *CODA*^73^. We reconstructed marginal distributions of net diversification rates for each branch in the BEAST chronogram using the posterior distribution of evolutionary parameters sampled by the reversible jump MCMC algorithm in BAMM.

In order to address the question that the observed iLDG pattern might be the result of an association between latitude and diversification rates, we compared their net-diversification rates using the geographical state speciation and extinction model (GoeSSE)^6^,^74^,^75^ in the R package *diversitree*^76^. We incorporated sampling fraction where 72% tropical, 65% temperate, and 100% of species occurring in both regions were included in our phylogeny. A model in which diversification was independent of geographic state using AIC model weights was rejected. Next we compared AIC model weights between the full 7 parameter model and one with 6 where between-region speciation was not estimated (sAB = 0). We conducted both a BI and ML estimation for the 6 free parameters (speciation in area A and B; extinction in area A and B; dispersal in area A and B). For the Bayesian inference we set the tuning parameter *w* to the distance between the 5% and 95% quantiles from the marginal distribution of a preliminary run, then ran 1e + 05 MCMC generations, sampling every 100^th^ generation, to obtain an estimate of the parameters.

## Results

### Phylogenetic relationships

The aligned dataset comprised 8,663 bp. Protein coding genes in general showed no insertions or deletions (indels), but we found an amino acid deletion (3 indels) in the CAD alignment in *Rhantus orbignyi*. The CAD sequences were therefore realigned and translated into AA-sequences for quality control, but no obvious pseudogenes were recognized. The *18S* and *16S* rRNA genes showed several single or multi base indels between regions of high nucleotide conservation. No indels were found in the *12S* rRNA gene. Within the Colymbetinae, most of the internal nodes were supported by bootstrap values > 80, or posterior probabilities > 0.95. With the exception of the clade containing *Melanodytes pustulatus*, the phylogenies inferred by the model based approaches were highly compatible with the MP analysis (Supplemental Material A, Figure S2). Colymbetinae was always recovered monophyletic with strong support. The cosmopolitan genus *Rhantus* was polyphyletic with strong support in all analyses.

### Divergence time estimation

The results of the BEAST analysis and details for each node (HPD intervals) are shown in Figure S3, median ages of each node are indicated in Fig. 2. After checking convergence of the runs, the molecular dating approach of the model incorporating substitution rates calculated for different beetle lineages of the *COI* gene under the uniform prior distribution was selected (Table S5). However, both divergence time estimations using normal and uniform distributions were mostly compatible. The optimal model recovered an origin of the Colymbetinae at the Palaeocene-Eocene border, approximately 56 million years ago (Ma) (HPD 68.6–44.6 Ma). Our divergence time estimates of the “southern” species of the *Rhantus suturalis* clade (HPD 3.6–8.0 Ma) (*R. bacchusi*, *R. ekari*, *R. dani*, *R. supranubicus*, *R. suturalis*) were mostly congruent with the findings of Balke *et al.*^33^ and Toussaint *et al.*^35^ (2.7–4.3 *versus* 1.5–4.7 Ma respectively).

### Historical biogeography and diversification rate analysis (LTT)

The results of the BioGeoBEARS analysis are shown in Fig. 2 and Table 3. The model receiving the strongest support from the different analyses performed in *BioGeoBEARS* was the time constrained model 3 DEC + J. The ancestral areas of extant Colymbetinae diving beetles were reconstructed to be the warm temperate to temperate Eastern Palaearctic and Australian regions. Our results suggest that extant Colymbetinae persisted in the Eastern Palaearctic for 20 to 25 million years (Myr), and that modern lineages are a result of continuous colonization events from these ancestral areas. The results of the diversification rate analyses are shown in Table S6 and Figure S4. Neither the TreePar nor BAMM analysis detected a significant shift in diversification rate, therefore supporting a constant rate of diversification throughout the evolution of the group.

The results of the GeoSSE show that the full model was favoured over the model where the geographic range was independent of diversification. The 6 parameter model was favoured over the full 7 parameter model where between-region speciation was estimated (Table S7).

The results of the Bayesian analyses indicate that the different parameters were distinct (not overlapping 95% credible intervals) except for extinction. Net-diversification between tropical and temperate species also overlapped between their 95% credible intervals (Figure S5).

### Predicted Niche Occupancy (PNO) and Niche overlap

We used the results from the PNO profiles to calculate the niche overlap between biogeographical regions. The Pacific region stood out as having low niche overlap with the other regions, except for BIO3 and BIO4, which showed a different pattern. These two variables show low niche overlap between the Palaearctic and the Pacific, Neotropics, Afrotopics and Australian regions. The combined niche overlap showed a similar pattern with low overlap between the Pacific, Neotropics, Afrotropics and Australian regions with the Palaearctic regions (Figures S6 and S7).

### Testing for Phylogenetic Niche Conservatism (PNC)

For all bioclimatic variables, Blomberg’s K-value showed significant phylogenetic signal (*p* < 0.05) (Table S8). Among models of trait evolution, the OU model was selected as the best fitting model given the dAICc statistic for all bioclimatic variables (Table S9). We used the OU model, instead of the BM model, to reconstruct ancestral climate preferences using a modified version of the phytools function ‘*contMap*’ (Fig. 3).

## Discussion

**Figure 1:**
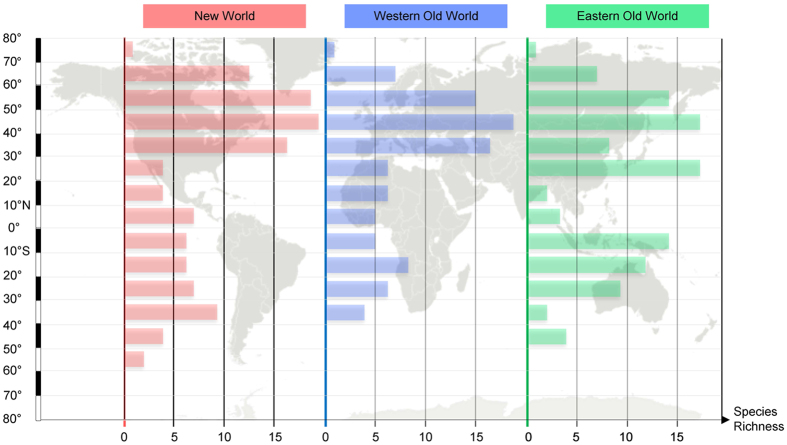
Inversed Latitudinal Diversity Gradient (iLDG) for species richness of Colymbetinae diving beetles. Species richness is declining towards the equator (red dotted line). The many species endemic to single mountain tops in the Eastern Old World cause an extratropical diversity peak. Species data was compiled from the world catalogue of Dytiscidae by^36^. Map (from Wikipedia) and species richness graphs were created using Microsoft Power Point 2010.

**Figure 2:**
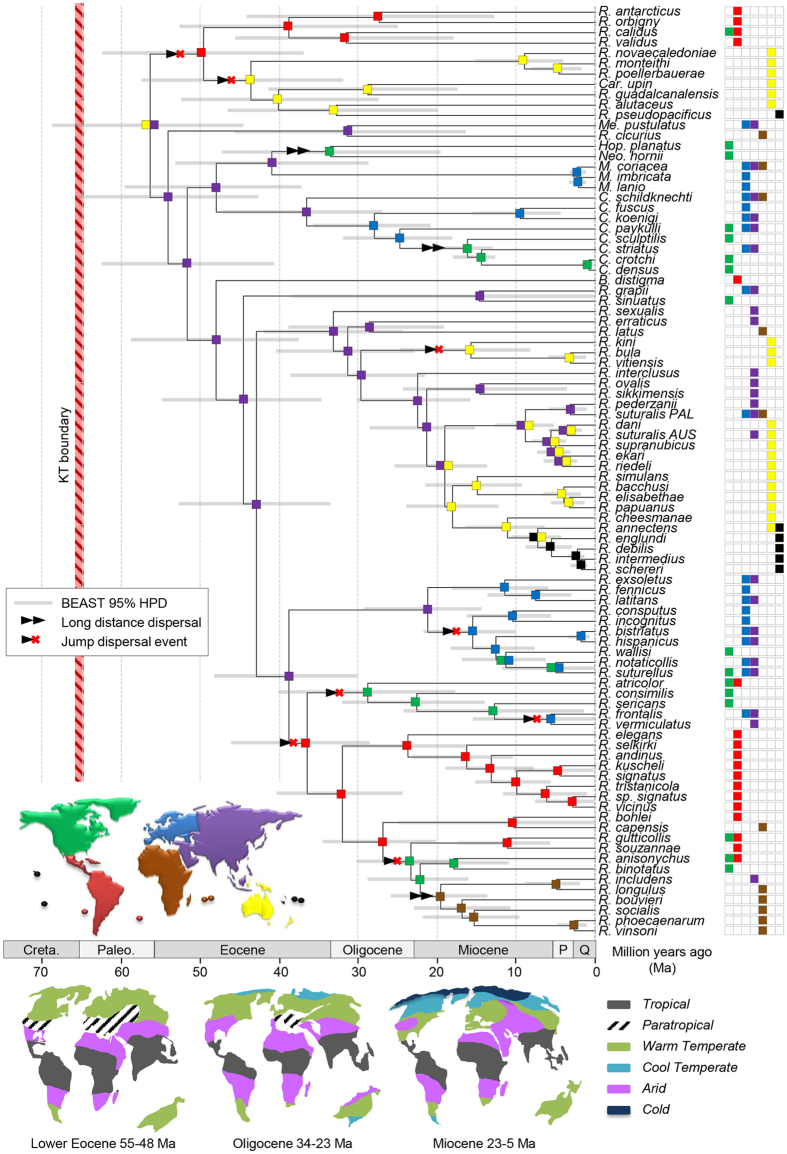
Temporal and biogeographical reconstruction of the Colymbetinae. We applied the BI topology as a starting tree in the BEAST analysis. The BioGeoBEARS approach was used to calculate the most probable ancestral biogeographical region at each node. 95% HPD intervals are indicated as bars at each node. Paleo climatic conditions (adapted by^77^) are illustrated for the Lower Eocene, Oligocene and Miocene in the lower part of the figure. Maps (from Wikipedia and the work of 77) were adapted, redrawn and colorized using Microsoft Power Point 2010.

**Figure 3:**
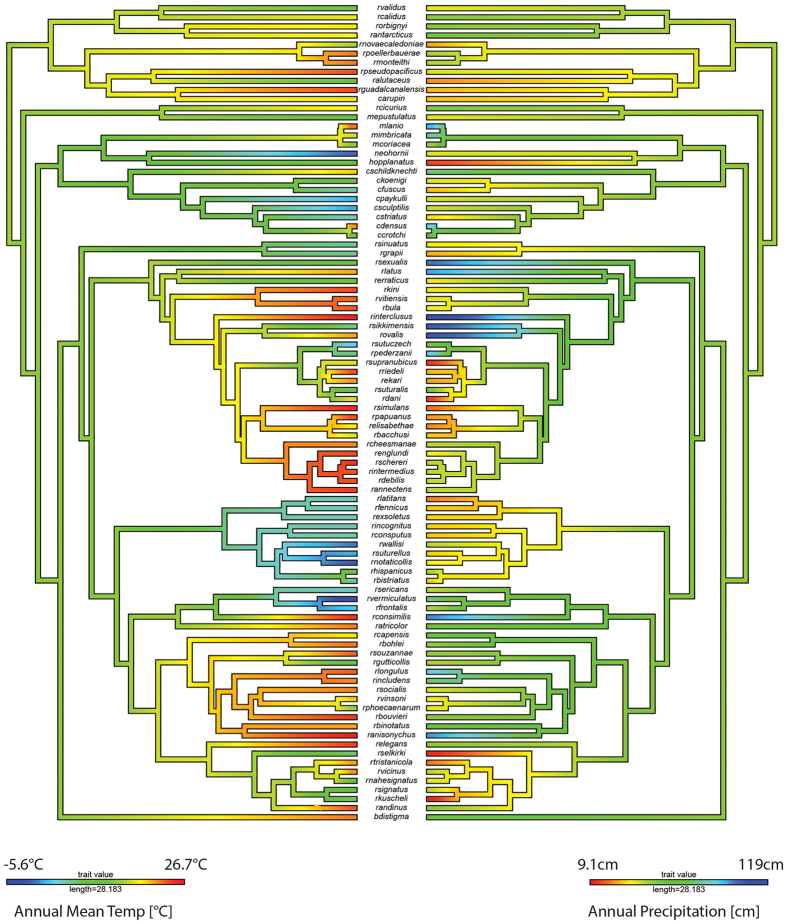


While many animal and plant taxa show increasing species richness towards the Equator, Colymbetinae exhibit a different pattern–they are most diverse in temperate areas (Fig. 1). In this study, we investigate if this pattern was a recent switch or if the group historically originated and diversified in temperate zones. The results of the biogeographical reconstruction indicate an Eastern Palaearctic and Australian origin of Colymbetinae at the Palaeocene-Eocene boundary, approximately 56 million years ago (Fig. 2). Since at least the late Jurassic, the climate of these areas were mostly temperate or warm temperate (Fig. 2)^77^. Thus, the biogeographical reconstruction infers temperate or warm temperate climate niches for the ancestral taxa. This niche was also inferred by the climate ancestral state reconstructions (Fig. 3). However, the inferred ancestral state is only one piece of information towards understanding phylogenetic niche conservatism, as we also need to know how conserved these traits are. For instance, a rapidly shifting ancestral climate preference indicates that this trait is not conserved and may have little to do with limiting where a taxon can reside. In contrast, if the preference is conserved beyond that expected under descent with modification, the ancestral state’s lability may strongly influence the evolutionary history of a clade and its diversity and distribution patterns (e.g.^65^,^66^,^78^). Instead, the ancestral state may not be representative for later climate preferences in a clade in which these preferences shift rapidly. The reason ancestral niche conservatism is important is because of the exponential accumulation of lineages through time even under a constant speciation model^79^,^80^. So the under PNC older nodes have a propagative effect, disproportionally contributing to current niche preferences. In order to test our hypothesis that the iLDG pattern in Colymbetinae is the result of ancestral temperate origin in combination with niche conservatism, we tested for PNC using standard methods as in Wiens *et al.*^66^ and Pyron *et al.*^65^. This is supported by the results of our analyses, as a significant amount of phylogenetic signal with additional support of the OU model is generally interpreted as an indication of trait conservatism^65^,^66^,^78^. Thus, we infer the ancestral area with its associated climatic conditions in combination with PNC as the underlying factors largely responsible for the observed iLDG pattern in Colymbetinae.

The importance of niche conservatism in various aspects of ecology, evolution and diversification was recently reviewed by Wiens *et al.*^66^. Biologists have perhaps disproportionally focused on examples of adaptation and (rapid) change, while the tendency among species to retain similar traits over long periods of time may be a factor just as important to understand diversity-distribution patterns. Wiens *et al.*^66^ broadly defined niche conservatism as “the retention of niche-related ecological traits over time”, applying whenever phylogenetic signal is stronger than expected under a pure BM model. A narrower concept of phylogenetic niche conservatism proposes that closely related species are ecologically *more* similar than would be expected under BM inheritance of traits with genetic drift (Losos 2008^81^). The preference of the OU model over the BM model for Colymbetinae serves as partial evidence for phylogenetic niche conservatism. However, observing a pattern of phylogenetic niche conservatism may have several explanations, such as evolutionary constraints from various factors (physiological, host choice, developmental and genetic), stabilizing selection or “phylogenetic inertia” (Cooper *et al.* 2010^82^). Our biogeographical reconstruction highlights multiple colonization and long distance dispersal events into the Indomalayan region out of the Palaearctic (*Rhantus pacificus* and *R. suturalis* clade). These results also indicate that the Neotropical clade containing *Rhantus calidus* originated as a result of colonization out of the Australian region via LDD or transitions through Antarctica. Transitions throughout Antarctica and Australia into other temperate regions appear plausible for highly vagile organisms because climatic conditions were temperate to cold temperate until the onset of the Oligocene^83^. Colymbetinae qualify as a highly vagile taxa; for example, the Neotropical species *Rhantus signatus* has colonized the island of Tristan da Cunha over 3500 km into the Atlantic^34^, suggesting long distance dispersal seems plausible. According to our analyses, the Afrotropical region was colonized twice by species of the *Rhantus bohlei* clade from the Nearctic and Neotropical regions (Fig. 2). The results indicate that *Rhantus capensis* colonized the Afrotropical region via long distance dispersal out of the Neotropics, whereas the remaining Afrotropical members of the clade are a result of long distance dispersal out of the Nearctic region. When present in tropical regions, Colymbetinae are mostly restricted to high altitude habitats and subalpine biomes^33^,^34^. Forty species can be found in the well sampled Indomalayan-Australasian archipelago, including thirty endemics, resulting in a peak of diversity in this otherwise temperate group. Most of these species are restricted to single high valleys or mountain tops, which are the sole regions in tropical latitudes in which low temperatures predominate^84^. The niche overlap results demonstrate that these habitats are roughly similar to the temperate regions. Adaptations to temperate climates would facilitate the colonization of tropical cool highlands. Similar processes might also explain the origin of the tropical lineages within the Agabinae, the sister group of Colymbetinae. While being mostly restricted to temperate regions, the genus *Agabus* also inhabits Afrotropical mountain ranges including the Cape region of South Africa with Mediterranean climate and has a small radiation in the high altitude areas of Ethiopia. The genus *Platynectes* inhabits the montane regions of the Andes. In the latter case, as well as in lineages of the Oriental and Australian *Platynectes*, lowland lineages are also known, but the phylogenetic relationships and their ancestral origin is not currently known at present^37^.

Our results suggest that the physiological niche conservatism hypotheses for high species diversity in the tropics can be equally applicable to explaining high temperate species richness^9^,^18^. Explanations for high species diversity in temperate regions, for groups that originated in temperate zones could also be explained by the hypotheses we discussed attempting to explain tropical biodiversity gradients based on their geological age and physiological niche adaptation of species. The ancestral biogeographical and environmental preference reconstructions results indicate that niche conservatism was largely responsible for restricting the distribution of the Colymbetinae.

While the vast majority of Earth’s biodiversity exhibits a gradient of species richness declining with increasing latitude^3^, others manifest the opposite pattern of biodiversity. However, the appearance of this inverted pattern of species diversity is likely to be the consequence of multiple interleaved processes of evolution. For Colymbetinae diving beetles, the most important processes inferred here are captured by the general theories of the centre-of-origin^20^, the time-for-speciation-effect^19^ and niche conservatism (reviewed in ref. 66). The generality of these conclusions remains to be tested by forthcoming empirical studies on other groups of organisms showing a clear iLDG pattern. But as these processes and effects are of a very general nature we would not be surprised if they turn out to be mechanisms governing iLDG patterns also in a range of other groups.

## Additional Information

**How to cite this article**: Morinière, J. *et al.* Phylogenetic niche conservatism explains an inverse latitudinal diversity gradient in freshwater arthropods. *Sci. Rep.* **6**, 26340; doi: 10.1038/srep26340 (2016).

## Supplementary Material

Supplementary Information

## Figures and Tables

**Table 1:** Adjacency matrix and dispersal probabilities within the different models tested.

	Adjecency matrix																				
	NA	SA	WPA	EPA	AFR	AUS	PAC														
NA	1	1	1	1	0	0	1														
SA	1	1	0	1	0	0	1														
WPA	1	0	1	1	1	0	0														
EPA	1	1	1	1	1	0	0														
AFR	0	0	1	1	1	1	0														
AUS	0	0	0	0	1	1	1														
PAC	1	1	0	0	0	1	1														

	Model 1 (M1)							Model 2 (M2)							Model 3 (M3)						
0–5 ma	NA	SA	WPA	EPA	AFR	AUS	PAC	NA	SA	WPA	EPA	AFR	AUS	PAC	NA	SA	WPA	EPA	AFR	AUS	PAC
	1	1	0.1	0.1	0.1	0.1	0.1	1	1	0.1	0	0	0	0.01	1	1	0.5	0	0	0	0.2
	1	1	0.1	0.1	0.1	0.1	0.1	1	1	0	0.01	0	0	0.1	1	1	0	0.3	0	0	0.1
	0.1	0.1	1	0.75	1	0.1	0.1	0.1	0	1	0.5	0.5	0	0	0.5	0	1	0.7	0.8	0	0
	0.1	0.1	0.75	1	0.75	0.1	0.1	0	0.01	0.5	1	0.01	0	0	0	0.3	0.7	1	0.3	0	0
	0.1	0.1	1	0.75	1	0.75	0.1	0	0	0.5	0.01	1	0.1	0.1	0	0	0.8	0.3	1	0.8	0.5
	0.1	0.1	0.1	0.1	0.75	1	0.25	0	0	0	0	0.1	1	0.1	0	0	0	0	0.8	1	0.5
	0.1	0.1	0.1	0.1	0.1	0.25	1	0.01	0.1	0	0	0.1	0.1	1	0.2	0.1	0	0	0.5	0.5	1
5–30 ma	1	0.75	0.1	0.1	0.1	0.1	0.1	1	0.5	0.1	0	0	0	0.01	1	0.8	0.5	0	0	0	0.2
	0.75	1	0.1	0.1	0.1	0.1	0.1	0.5	1	0	0.01	0	0	0.1	0.8	1	0	0.3	0	0	0.1
	0.1	0.1	1	0.75	0.75	0.1	0.1	0.1	0	1	0.5	0.5	0	0	0.5	0	1	0.7	0.8	0	0
	0.1	0.1	0.75	1	0.75	0.1	0.1	0	0.01	0.5	1	0.01	0	0	0	0.3	0.7	1	0.3	0	0
	0.1	0.1	0.75	0.75	1	0.75	0.1	0	0	0.5	0.01	1	0.1	0.1	0	0	0.8	0.3	1	0.8	0.5
	0.1	0.1	0.1	0.1	0.75	1	0.25	0	0	0	0	0.1	1	0.1	0	0	0	0	0.8	1	0.5
	0.1	0.1	0.1	0.1	0.1	0.25	1	0.01	0.1	0	0	0.1	0.1	1	0.2	0.1	0	0	0.5	0.5	1
30–45 ma	1	0.75	0.75	0.1	0.1	0.1	0.1	1	0.5	0.1	0	0	0	0.01	1	0.6	0.6	0	0	0	0.15
	0.75	1	0.1	0.1	0.1	0.1	0.1	0.5	1	0	0.01	0	0	0.1	0.6	1	0	0.5	0	0	0.1
	0.75	0.1	1	0.5	0.5	0.1	0.1	0.1	0	1	0.5	0.5	0	0	0.6	0	1	0.6	0.7	0	0
	0.1	0.1	0.5	1	0.5	0.1	0.1	0	0.01	0.5	1	0.01	0	0	0	0.5	0.6	1	0.4	0	0
	0.1	0.1	0.5	0.5	1	0.5	0.1	0	0	0.5	0.01	1	0.1	0.1	0	0	0.7	0.4	1	0.6	0
	0.1	0.1	0.1	0.1	0.5	1	0.25	0	0	0	0	0.1	1	0.1	0	0	0	0	0.6	1	0.5
	0.1	0.1	0.1	0.1	0.1	0.25	1	0.01	0.1	0	0	0.1	0.1	1	0.15	0.1	0	0	0	0.5	1
45–70 ma	1	0.25	0.75	0.1	0.5	0.1	0.1	1	0.5	0.1	0	0	0	0.01	1	0.4	0.4	0	0	0	0.1
	0.25	1	0.1	0.1	0.1	0.5	0.5	0.5	1	0	0.01	0	0	0.1	0.4	1	0	0.7	0	0	0.1
	0.75	0.1	1	0.25	0.25	0.1	0.1	0.1	0	1	0.5	0.5	0	0	0.4	0	1	0.5	0.6	0	0
	0.1	0.1	0.25	1	0.25	0.1	0.1	0	0.01	0.5	1	0.01	0	0	0	0.7	0.5	1	0.5	0.1	0
	0.5	0.1	0.25	0.25	1	0.1	0.1	0	0	0.5	0.01	1	0.1	0.1	0	0	0.6	0.5	1	0.4	0
	0.1	0.5	0.1	0.1	0.1	1	0.1	0	0	0	0	0.1	1	0.1	0	0	0	0.1	0.4	1	0.5
	0.1	0.5	0.1	0.1	0.1	0.1	1	0.01	0.1	0	0	0.1	0.1	1	0.1	0.1	0	0	0	0.5	1

Abbreviations: NA = Nearctic, SA = Neotropics, WPA = Western Palaearctic, EPA = Eastern Palaearctic, AFR = Afrotropics, AUS = Australis, PAC = Pacific region.

**Table 2:** Bioclimatic variables (WorldClim - http://www.worldclim.org) used in this study.

**BIO1** – Annual Mean Temperature
**BIO2** – Mean Diurnal Range (monthly mean of max Temp – min Temp)
**BIO3** – Isothermality (Mean Diurnal Range/Temperature Annual Range*100)
**BIO4** – Temperature Seasonality (standard deviation*100)
**BIO12** – Annual Precipitation
**BIO15** – Precipitation Seasonality (Coefficient of Variation)

**Table 3:** Results of the *BioGeoBEARS* analyses performed.

MODEL	number of free parameters	LnL Results	AICc value	AICc weights	Relative model probabilities based on AICc
DEC_M1m_time	2	−182.6851307	369.508193	1.2082E − 06	0%
DEC + J_M1m_time	3	−167.9881476	342.255365	1.00E + 00	99%
DIVALIKE_M1m_time	2	−189.7320789	383.602089	1.05E − 09	0%
DIVALIKE + J_M1m_time	3	−174.2836319	354.846334	1.84E − 03	0%
BAYAREALIKE_M1m_time	2	−201.4968909	407.131713	8.1711E − 15	0%
BAYAREALIKE + J_M1m_time	3	−175.8161016	357.911273	3.98E − 04	0%
DEC_M2_time	2	−189.075381	431.446498	4.2894E − 20	0%
DEC + J_M2_time	3	−179.4268355	414.651899	1.90E − 16	0%
DIVALIKE_M2_time	2	−197.8204624	450.7026	2.8248E − 24	0%
DIVALIKE + J_M2_time	3	−187.7674918	432.119206	3.0642E − 20	0%
BAYAREALIKE_M2_time	2	−204.3778223	448.375322	9.0437E − 24	0%
BAYAREALIKE + J_M2_time	3	−185.7615947	393.047963	9.3439E − 12	0%
DEC_M3m_time	2	−183.4127287	377.426133	2.3055E − 08	0%
DEC + J_M3m_time	3	−173.6543751	361.506569	6.6017E − 05	0%
DIVALIKE_M3m_time	2	−187.3947768	390.544831	3.2664E − 11	0%
DIVALIKE + J_M3m_time	3	−178.7766499	375.234088	6.8986E − 08	0%
BAYAREALIKE_M3m_time	2	−200.5770228	414.269764	2.3029E − 16	0%
BAYAREALIKE + J_M3m_time	3	−176.9474415	368.710021	1.8007E − 06	0%
DEC_time	2	−213.6542834	431.446498	4.2894E − 20	0%
DEC + J_time	3	−204.1864144	414.651832	1.90E − 16	0%
DIVALIKE_time	2	−223.2823343	450.702597	2.82E − 24	0%
DIVALIKE + J_time	3	−212.9200679	432.11952	3.06E − 20	0%
BAYAREALIKE_time	2	−222.1186952	448.375341	9.0436E − 24	0%
BAYAREALIKE + J_time	3	−193.3844468	393.047961	9.3439E − 12	0%
DEC_adj	2	−186.6441009	382.288693	2.0271E − 09	0%
DEC + J_adj	3	−177.6137495	365.132741	1.08E − 05	0%
DIVALIKE_adj	2	−193.2034499	399.778856	3.228E − 13	0%
DIVALIKE + J_adj	3	−184.4775093	381.814053	2.57E − 09	0%
BAYAREALIKE_adj	2	−205.0659164	412.893576	4.5826E − 16	0%
BAYAREALIKE + J_adj	3	−181.2154758	377.802259	1.9102E − 08	0%
DEC	2	−213.6542833	370.963388	5.8362E − 07	0%
DEC + J	3	−204.1863811	353.58782	3.46E − 03	0%
DIVALIKE	2	−223.2823332	378.927485	1.09E − 08	0%
DIVALIKE + J	3	−212.9202253	363.83237	2.06E−05	0%
BAYAREALIKE	2	−222.1187049	405.291977	2.0501E − 14	0%
BAYAREALIKE + J	3	−193.3844454	360.173953	0.00012854	0%

M1 − 3m_time = time stratified with adjacency matrix and manual dispersal multipliers.

adj = just using adjacency matrix.

time = time stratified with just manual dispersal multipliers.

## References

[b1] Bates H. W. The Naturalist on the River Amazons: A Record of Adventures, Habits of Animals, Sketches of Brazilian and Indian Life, and Aspects of Nature Under the Equator, During Eleven Years of Travel. With a Memoir of the Author, by *Edward Clodd*. *J. Murray* (1892).

[b2] Condamine F. L., Sperling F. A., Wahlberg N., Rasplus J. Y. & Kergoat G. J. What causes latitudinal gradients in species diversity?Evolutionary processes and ecological constraints on swallowtail biodiversity. Ecol. Let. 15(3), 267–277 (2012). 22251895 10.1111/j.1461-0248.2011.01737.x

[b3] Hillebrand H. On the generality of the latitudinal diversity gradient. Am. Nat. 163(2), 192–211 (2004). 14970922 10.1086/381004

[b4] Mittelbach G. G. *et al.* Evolution and the latitudinal diversity gradient: speciation, extinction and biogeography. Ecol. Let. 10(4), 315–331 (2007). 17355570 10.1111/j.1461-0248.2007.01020.x

[b5] Pianka E. R. Latitudinal gradients in species diversity: a review of concepts. Am. Nat. 910(100) 33–46 (1966).

[b6] Rolland J., Condamine F. L., Jiguet F. & Morlon H. Faster speciation and reduced extinction in the tropics contribute to the mammalian latitudinal diversity gradient. PLoS Biol. 12(1), e1001775 (2014). 24492316 10.1371/journal.pbio.1001775 PMC3904837

[b7] Weir J. T. & Schluter D. The latitudinal gradient in recent speciation and extinction rates of birds and mammals. Science. 315(5818), 1574–1576 (2007). 17363673 10.1126/science.1135590

[b8] Willig M. R., Kaufman D. M. & Stevens R. D. Latitudinal gradients of biodiversity: pattern, process, scale, and synthesis. Annu. Rev. Ecol. Evol. Syst. 34, 273–309 (2003).

[b9] Visser V., Clayton W. D., Simpson D. A., Freckleton R. P. & Osborne C. P. Mechanisms driving an unusual latitudinal diversity gradient for grasses. Global Ecol. Biogeogr. 23(1), 61–75 (2014).

[b10] McKenna D. D. & Farrell B. D. Tropical forests are both evolutionary cradles and museums of leaf beetle diversity. PNAS. 103(29), 10947–10951 (2006). 16818884 10.1073/pnas.0602712103 PMC1544154

[b11] Stebbins G. L. Flowering plants: evolution above the species level. (E. Arnold, London 1974).

[b12] Chown S. L. & Gaston K. J. Areas, cradles and museums: the latitudinal gradient in species richness. Trends Ecol. Evol. 15(8), 311–315 (2000). 10884694 10.1016/s0169-5347(00)01910-8

[b13] Fischer A. G. Latitudinal variations in organic diversity. Evolution 14(1), 64–81 (1960).

[b14] Jablonski D., Roy K. & Valentine J. W. Out of the tropics: evolutionary dynamics of the latitudinal diversity gradient. Science. 314(5796), 102–106 (2006). 17023653 10.1126/science.1130880

[b15] Kerkhoff A. J., Moriarty P. E. & Weiser M. D. The latitudinal species richness gradient in New World woody angiosperms is consistent with the tropical conservatism hypothesis. PNAS. 111(22), 8125–8130 (2014). 24847062 10.1073/pnas.1308932111 PMC4050539

[b16] Crisp M. D. & Cook L. G. Phylogenetic niche conservatism: what are the underlying evolutionary and ecological causes? New Phytol 196(3), 681–694 (2012). 22943495 10.1111/j.1469-8137.2012.04298.x

[b17] Wiens J. J. & Donoghue M. J. Historical biogeography, ecology and species richness. Trends Ecol, Evol. 19(12), 639–644 (2004). 16701326 10.1016/j.tree.2004.09.011

[b18] Zanne A. E. *et al.* Three keys to the radiation of angiosperms into freezing environments. Nature 506(7486), 89–92 (2014). 24362564 10.1038/nature12872

[b19] Stephens P. R. & Wiens J. J. Explaining species richness from continents to communities: the time‐for‐speciation effect in emydid turtles. Am. Nat. 161(1), 112–128 (2003). 12650466 10.1086/345091

[b20] Ricklefs R. E. & Schluter D. Species diversity in ecological communities: historical and geographical perspectives. (University of Chicago Press, 1993).

[b21] Rohde K. Latitudinal gradients in species diversity: the search for the primary cause. 514–527 (Oikos, 1992).

[b22] Mannion P. D., Upchurch P., Benson R. B. & Goswami A. The latitudinal biodiversity gradient through deep time. Trends Ecol. Evol. 29(1), 42–50 (2014). 24139126 10.1016/j.tree.2013.09.012

[b23] Wiens J. J., Sukumaran J., Pyron R. A. & Brown R. M. Evolutionary and biogeographic origins of high tropical diversity in Old World frogs (Ranidae). Evolution 63(5), 1217–1231 (2009). 19154386 10.1111/j.1558-5646.2009.00610.x

[b24] Cook R. E. Variation in species density of North American birds. Syst. Biol. 18(1), 63–84 (1969).

[b25] Gauld I. D. Latitudinal gradients in ichneumonid species richness in Australia. Ecol. Entomol 11(2), 155–161 (1986).

[b26] Hawkins B. A. Pattern and process in host-parasitoid interactions. (Cambridge University Press, 1994).

[b27] Janzen D. H. The peak in North American ichneumonid species richness lies between 38 degrees and 42 degrees N. Ecology 532–537 (1981).

[b28] Owen D. F. & Owen J. Species diversity in temperate and tropical Ichneumonidae. Nature. 249(5457), 583–584 (1974).

[b29] Santelices B. Phytogeographic characterization of the temperate coast of Pacific South America. Phycologia 19(1), 1–12 (1980).

[b30] Dixon A. F. G., Kindlmann P., Leps J. & Holman J. Why there are so few species of aphids, especially in the tropics. Am. Nat. 129, 580–592 (1987).

[b31] Kindlmann P., Schödelbauerová I. & Dixon A. F. Inverse latitudinal gradients in species diversity. Scaling biodiversity. 246–257 (Cambridge University Press, 2007).

[b32] Smith S. A., Stephens P. R. & Wiens J. J. Replicate patterns of species richness, historical biogeography, and phylogeny in Holarctic treefrogs. Evolution. 59(11), 2433–2450 (2005). 16396184

[b33] Balke M. *et al.* New Guinea highland origin of a widespread arthropod supertramp. Proc. Soc. London ,Ser. B. 276, 2359–2367 (2009). 10.1098/rspb.2009.0015 PMC2690458 19364747

[b34] Morinière J. *et al.* Anisomeriini diving beetles–an Atlantic–Pacific Island disjunction on Tristan da Cunha and Robinson Crusoe Island, Juan Fernández? Cladistics 31(2), 166–176 (2015). 10.1111/cla.12074 34758583

[b35] Toussaint E. F., Sagata K., Surbakti S., Hendrich L. & Balke M. Australasian sky islands act as a diversity pump facilitating peripheral speciation and complex reversal from narrow endemic to widespread ecological supertramp. Ecol. Evol. 3(4), 1031–1049 (2013). 23610642 10.1002/ece3.517 PMC3631412

[b36] Nilsson A. N. A world catalogue of the family Dytiscidae, or the diving beetles (Coleoptera, Adephaga). (Umeå, 2013).

[b37] Ribera I., Nilsson A. N. & Vogler A. P. Phylogeny and historical biogeography of Agabinae diving beetles (Coleoptera) inferred from mitochondrial DNA sequences. Mol. Phylogenet Evol. 30, 545–562 (2004). 15012938 10.1016/S1055-7903(03)00224-0

[b38] Tänzler R., Toussaint E. F., Suhardjono Y. R., Balke M. & Riedel A. Multiple transgressions of Wallace’s Line explain diversity of flightless Trigonopterus weevils on Bali. Proc. Soc. London, Ser. B. 281(1782), 20132528 (2014). 10.1098/rspb.2013.2528 PMC3973253 24648218

[b39] Maddison W. P. & Maddison D. R. Mesquite: a modular system for evolutionary analysis. Version 3.04, http://mesquiteproject.org. (2015).

[b40] Goloboff P. A., Farris J. S. & Nixon K. C. TNT, a free program for phylogenetic analysis. Cladistics. 24(5), 774–786 (2008).

[b41] Silvestro D. & Michalak I. raxmlGUI: a graphical front-end for RAxML. Org. Divers. Evol. 12(4), 335–337 (2012).

[b42] Lanfear R., Calcott B., Ho S. Y. & Guindon S. PartitionFinder: combined selection of partitioning schemes and substitution models for phylogenetic analyses. Mol. Biol. Evol. 29(6), 1695–1701 (2012). 22319168 10.1093/molbev/mss020

[b43] Ronquist F. *et al.* MrBayes 3.2: efficient Bayesian phylogenetic inference and model choice across a large model space. Syst. Biol. 61(3), 539–542 (2012). 22357727 10.1093/sysbio/sys029 PMC3329765

[b44] Drummond A. J. & Rambaut A. BEAST: Bayesian evolutionary analysis by sampling trees. BMC Evol. Biol. 7(1), 214 (2007). 17996036 10.1186/1471-2148-7-214 PMC2247476

[b45] Toussaint E. F. *et al.* The towering orogeny of New Guinea as a trigger for arthropod megadiversity. Nat. comm. doi: 10.1038/ncomms5001 (2014). 24874774

[b46] Drummond A. J., Suchard M. A., Xie D. & Rambaut A. Bayesian phylogenetics with BEAUti and the BEAST 1.7. Mol. Biol. Evol. 29(8), 1969–1973 (2012). 22367748 10.1093/molbev/mss075 PMC3408070

[b47] Matzke N. J. *BioGeoBEARS*: BioGeography with Bayesian (and likelihood) evolutionary analysis in R Scripts. *R package ‘BioGeoBEARS’. URL:* http://CRAN.R-project.org/package=BioGeoBEARS (2013).

[b48] Matzke N. J. Model selection in historical biogeography reveals that founder-event speciation is a crucial process in island clades. Syst. Biol. 63(6), 951–970 (2014). 25123369 10.1093/sysbio/syu056

[b49] Ree R. H. Detecting the historical signature of key innovations using stochastic models of character evolution and cladogenesis. Evolution 59(2), 257–265 (2005). 15807412

[b50] Ree R. H. & Smith S. A. Maximum likelihood inference of geographic range evolution by dispersal, local extinction, and cladogenesis. Syst. Biol. 57(1), 4–14 (2008). 18253896 10.1080/10635150701883881

[b51] Ronquist F. Dispersal-vicariance analysis: a new approach to the quantification of historical biogeography. Syst. Biol. 46(1), 195–203 (1997).

[b52] Landis M. J., Matzke N. J., Moore B. R. & Huelsenbeck J. P. Bayesian analysis of biogeography when the number of areas is large. Syst. Biol. doi: 10.1093/sysbio/syt040 (2013). PMC4064008 23736102

[b53] Miller K. G. *et al.* The Phanerozoic record of global sea-level change. Science. 310(5752), 1293–1298 (2005). 16311326 10.1126/science.1116412

[b54] Seton M., Müller R. D., Zahirovic S., Gaina C., Torsvik T. & Shephard G. *et al.* Global continental and ocean basin reconstructions since 200Ma. Earth Sci. Rev. 113(3), 212–270 (2012).

[b55] Hernandez P. A., Graham C. H., Master L. L. & Albert D. L. The effect of sample size and species characteristics on performance of different species distribution modeling methods. Ecography 29(5), 773–785 (2006).

[b56] Evans M. E., Smith S. A., Flynn R. S. & Donoghue M. J. Climate, Niche Evolution, and Diversification of the “Bird‐Cage” Evening Primroses (Oenothera, Sections Anogra and Kleinia). Am. Nat. 173(2), 225–240 (2009). 19072708 10.1086/595757

[b57] Hawlitschek O., Porch N., Hendrich L. & Balke M. Ecological niche modelling and nDNA sequencing support a new, morphologically cryptic beetle species unveiled by DNA barcoding. PLoS One. 6(2), 1–14 (2011). 10.1371/journal.pone.0016662 PMC3036709 21347370

[b58] Hawlitschek O. *et al.* Pleistocene climate change promoted rapid diversification of aquatic invertebrates in Southeast Australia. BMC Evol. Biol. 12(1), 142 (2012). 22873814 10.1186/1471-2148-12-142 PMC3503846

[b59] Elith J. *et al.* A statistical explanation of *Maxent* for ecologists. Divers. Distrib. 17(1), 43–57 (2011).

[b60] Phillips S. J. & Dudík M. Modeling of species distributions with *Maxent*: new extensions and a comprehensive evaluation. Ecography. 31(2), 161–175 (2008).

[b61] Pearson R. G., Raxworthy C. J., Nakamura M. & Townsend Peterson A. Predicting species distributions from small numbers of occurrence records: a test case using cryptic geckos in Madagascar. J. Biogeogr. 34(1), 102–117 (2007).

[b62] Hijmans R. J. & van Etten J. *raster*: Geographic analysis and modeling with raster data. *R package ‘raster’. URL:* http://cran.r-project.org/web/packages/raster.pdf (2012).

[b63] Heibl C., Calenge C. & Heibl M. C. R package ‘phyloclim’. URL: http://cran.r-project.org/web/packages/phyloclim/index.html (2013).

[b64] Warren D. L., Glor R. E. & Turelli M. ENMTools: a toolbox for comparative studies of environmental niche models. Ecography 33(3), 607–611 (2010).

[b65] Pyron R. A., Costa G. C., Patten M. A. & Burbrink F. T. Phylogenetic niche conservatism and the evolutionary basis of ecological speciation. Biol.Rev. 90(4), 1248–1262 (2014). 25428167 10.1111/brv.12154

[b66] Wiens J. J. *et al.* Niche conservatism as an emerging principle in ecology and conservation biology. Ecol. Let. 13(10), 1310–1324 (2010). 20649638 10.1111/j.1461-0248.2010.01515.x

[b67] Blomberg S. P., Garland T. Jr & Ives A. R. Testing for phylogenetic signal in comparative data: behavioral traits are more labile. Evolution 57(4), 717–745 (2003). 12778543 10.1111/j.0014-3820.2003.tb00285.x

[b68] Butler M. A. & King A. A. Phylogenetic comparative analysis: a modeling approach for adaptive evolution. Am. Nat. 164(6), 683–695 (2004). 10.1086/426002 29641928

[b69] Felsenstein J. Maximum likelihood and minimum-steps methods for estimating evolutionary trees from data on discrete characters. Syst. Zool. 240–249 (1973).

[b70] Kozak K. H. & Wiens J. J. Accelerated rates of climatic‐niche evolution underlie rapid species diversification. *Ecol*. Let. 13(11), 1378–1389 (2010a). 10.1111/j.1461-0248.2010.01530.x 20875038

[b71] Rabosky D. L. Automatic detection of key innovations, rate shifts, and diversity-dependence on phylogenetic trees. PLoS One. 9(2), e89543 (2014a). 24586858 10.1371/journal.pone.0089543 PMC3935878

[b72] Rabosky D. L. *et al.* *BAMMtools*: an R package for the analysis of evolutionary dynamics on phylogenetic trees. Methods Ecol. Evol. 5(7), 701–707 (2014b).

[b73] Plummer M., Best N., Cowles K. & Vines K. CODA: Convergence diagnosis and output analysis for MCMC. R news. 6(1), 7–11 (2006).

[b74] Goldberg E. E., Lancaster L. T. & Ree R. H. Phylogenetic inference of reciprocal effects between geographic range evolution and diversification. Syst. Biol. 60(4), 451–465 (2011). 21551125 10.1093/sysbio/syr046

[b75] Rolland J., Condamine F. L., Beeravolu C. R., Jiguet F. & Morlon H. Dispersal is a major driver of the latitudinal diversity gradient of Carnivora. Global Ecol. Biogeogr. 24(9), 1059–1071 (2015).

[b76] FitzJohn R. G. Diversitree: comparative phylogenetic analyses of diversification in R. *Methods Ecol*. Evol. 3(6), 1084–1092 (2012).

[b77] Boucot A. J., Chen X. & Scotese C. R. Phanerozoic paleoclimate: An atlas of lithologic indicators of climate. Society of Economic Paleontologists and Mineralogists (2013).

[b78] Kozak K. H. & Wiens J. J. Niche Conservatism Drives Elevational Diversity Patterns in Appalachian Salamanders. Am. Nat. 176(1), 40–55 (2010b) 20497055 10.1086/653031

[b79] Nee S. Birth-death models in macroevolution. Annu. Rev. Ecol. Evol. Syst. 37, 1–17 (2006).

[b80] Ricklefs R. E. Estimating diversification rates from phylogenetic information. Trends Ecol. Evol. 22(11), 601–610 (2007). 17963995 10.1016/j.tree.2007.06.013

[b81] Losos J. B. Phylogenetic niche conservatism, phylogenetic signal and the relationship between phylogenetic relatedness and ecological similarity among species. Ecology letters 11(10), 995–1003 (2008). 18673385 10.1111/j.1461-0248.2008.01229.x

[b82] Cooper N., Jetz W. & Freckleton R. P. Phylogenetic comparative approaches for studying niche conservatism. Journal of evolutionary biology. 23(12), 2529–2539 (2010). 20964782 10.1111/j.1420-9101.2010.02144.x

[b83] Almeida E. A., Pie M. R., Brady S. G. & Danforth B. N. Biogeography and diversification of colletid bees (Hymenoptera: Colletidae): emerging patterns from the southern end of the world. J. Biogeogr. 39(3), 526–544 (2012).

[b84] Sarmiento G. Ecological features of climate in high tropical mountains. High altitude tropical biogeography, 11–45 (Oxord University Press, 1986).

