# Supplementary material for: Phylogenetic niche conservatism explains an inverse latitudinal diversity gradient in freshwater arthropods
Source: Sci Rep. 2016 May 24;6:26340. doi: 10.1038/srep26340 (PMC4877923; doi:10.1038/srep26340)
Supplement: Supplementary Information [file srep26340-s1.pdf]

Phylogenetic niche conservatism explains an inverse latitudinal diversity gradient in freshwater arthropods

Jérôme Morinière[1,*], Matthew H. Van Dam[1], Oliver Hawlitschek[1,2], Johannes Bergsten[3], Mariano C. Michat[4], Lars Hendrich[1], Ignacio Ribera[2], Emmanuel F.A. Toussaint[1, 5, #], Michael Balke[1, 6, #]

[1] *SNSB-Bavarian State Collection of Zoology, Münchhausenstrasse 21, 81247, Munich, Germany*
[2] *Institute of Evolutionary Biology (CSIC-Universitat Pompeu Fabra), Passeig Maritim de la Barceloneta 37, 08003 Barcelona, Spain*
[3] *Department of Zoology, Swedish Museum of Natural History, Box 50007, SE-10405, Stockholm, Sweden*
[4] *IBBEA, CONICET-UBA, Laboratory of Entomology-DBBE_FCEN, University of Buenos Aires, Buenos Aires, Argentina*
[5] *Department of Ecology & Evolutionary Biology & Division of Entomology, Biodiversity Institute, University of Kansas, Lawrence, KS 66045, USA*
[6] *GeoBioCenter, Ludwig-Maximilians-Universität München, Munich, Germany*

* Corresponding author: email address: Moriniere@zsm.mwn.de

[#] Joint Senior Authors

Supplementary Material:

Table S1 - GenBank accession numbers and BOLD process IDs

Taxa	SampleID	*12s*	*16s*	*cob*	*5'co1*	*3'co1*	*co2*	*18s*	*h4*	*ark*	*cad*	*eno*	*wng*	*ef1a*
Agabus sturmii (Gyllenhal, 1808)	JMAGA03	ZSMDB124-16	ZSMDB124-16	ZSMDB124-16	ZSMDB124-16	ZSMDB124-16	ZSMDB124-16	ZSMDB124-16	ZSMDB124-16	-----	-----	-----	ZSMDB124-16	-----
Agabus sp. Aethiopiae	JMAGA06	ZSMDB127-16	-----	ZSMDB127-16	ZSMDB127-16	ZSMDB127-16	-----	ZSMDB127-16	ZSMDB127-16	-----	-----	ZSMDB127-16	ZSMDB127-16	-----
Agabus conspersus (Marsham, 1802)	JMAGA08	ZSMDB129-16	ZSMDB129-16	ZSMDB129-16	ZSMDB129-16	ZSMDB129-16	ZSMDB129-16	ZSMDB129-16	ZSMDB129-16	-----	ZSMDB129-16	ZSMDB129-16	ZSMDB129-16	ZSMDB129-16
Agabus undulatus (Schrank, 1776)	JMAGA05	-----	-----	ZSMDB126-16	ZSMDB126-16	-----	ZSMDB126-16	-----	ZSMDB126-16	-----	-----	-----	ZSMDB126-16	-----
Ilybius ater (DeGeer, 1774)	JMAGA02	ZSMDB123-16	-----	ZSMDB123-16	ZSMDB123-16	ZSMDB123-16	ZSMDB123-16	ZSMDB123-16	-----	ZSMDB123-16	ZSMDB123-16	----	ZSMDB123-16	-----
Agabus freudeii Guéorguiev, 1975	JMAGA07	ZSMDB128-16	ZSMDB128-16	-----	ZSMDB128-16	ZSMDB128-16	ZSMDB128-16	ZSMDB128-16	-----	-----	ZSMDB128-16	ZSMDB128-16	ZSMDB128-16	ZSMDB128-16
Platynectes sp1	JMAGA14	ZSMDB135-16	-----	-----	ZSMDB135-16	ZSMDB135-16	ZSMDB135-16	ZSMDB135-16	-----	-----	-----	-----	ZSMDB135-16	ZSMDB135-16
Platynectes sp2	JMAGA09	ZSMDB130-16	ZSMDB130-16	-----	ZSMDB130-16	ZSMDB130-16	ZSMDB130-16	ZSMDB130-16	-----	-----	ZSMDB130-16	ZSMDB130-16	ZSMDB130-16	ZSMDB130-16
Platynectes sp3	JMAGA13	ZSMDB134-16	-----	-----	ZSMDB134-16	ZSMDB134-16	ZSMDB134-16	-----	-----	-----	-----	-----	ZSMDB134-16	-----
Agametrus sp.	JMAGA04	ZSMDB125-16	ZSMDB125-16	-----	ZSMDB125-16	ZSMDB125-16	ZSMDB125-16	ZSMDB125-16	-----	ZSMDB125-16	ZSMDB125-16	ZSMDB125-16	ZSMDB125-16	-----
Platynectes sp4	JMAGA15	-----	-----	-----	ZSMDB136-16	ZSMDB136-16	-----	-----	-----	-----	ZSMDB136-16	ZSMDB136-16	ZSMDB136-16	-----
Platynectes decemnotatus (Aubé, 1838)	JMAGA16	ZSMDB137-16	-----	-----	ZSMDB137-16	-----	-----	ZSMDB137-16	ZSMDB137-16	-----	-----	-----	ZSMDB137-16	ZSMDB137-16
Platynectes sp4	JMAGA17	-----	-----	-----	ZSMDB138-16	ZSMDB138-16	ZSMDB138-16	-----	-----	-----	-----	ZSMDB138-16	ZSMDB138-16	ZSMDB138-16
Platynectes sp5	JMAGA11	ZSMDB132-16	-----	ZSMDB132-16	ZSMDB132-16	ZSMDB132-16	ZSMDB132-16	ZSMDB132-16	-----	-----	-----	ZSMDB132-16	ZSMDB132-16	-----
Platynectes sp5	JMAGA12	-----	-----	-----	ZSMDB133-16	ZSMDB133-16	ZSMDB133-16	ZSMDB133-16	-----	-----	ZSMDB133-16	ZSMDB133-16	ZSMDB133-16	-----
Agametrus nitens Sharp, 1887	JMAGA01	-----	-----	ZSMDB122-16	-----	ZSMDB122-16	-----	-----	ZSMDB122-16	-----	-----	-----	ZSMDB122-16	-----
Platynectes sp6	JMAGA10	ZSMDB131-16	-----	-----	ZSMDB131-16	-----	ZSMDB131-16	ZSMDB131-16	-----	-----	-----	ZSMDB131-16	ZSMDB131-16	ZSMDB131-16
Bunites distigma (Brullé, 1837)	IRBD	-----	KJ637894	KJ637933	KJ637877	KJ637978	-----	KJ637914	ZSMDB004-14	-----	-----	-----	----	-----
Colymbetes crotchi Sharp, 1882	797741	ZSMDB083-16	ZSMDB083-16	ZSMDB083-16	-----	ZSMDB083-16	ZSMDB083-16	ZSMDB083-16	-----	ZSMDB083-16	ZSMDB083-16	ZSMDB083-16	ZSMDB083-16	-----
Colymbetes densus LeConte, 1859	797748	ZSMDB084-16	ZSMDB084-16	ZSMDB084-16	-----	ZSMDB084-16	ZSMDB084-16	ZSMDB084-16	-----	ZSMDB084-16	ZSMDB084-16	-----	ZSMDB084-16	-----
Colymbetes fuscus (Linnaeus, 1758)	MB0179	KJ637966	KJ637892	KJ637931	KJ637875	KJ637976	KJ637989	KJ637911	ZSMDB005-14	-----	KJ638046	KJ638067	KJ638022	KJ637949
Colymbetes koenigi Zaitsev, 1927	MB0195	ZSMDB079-16	ZSMDB079-16	ZSMDB079-16	ZSMDB079-16	ZSMDB079-16	ZSMDB079-16	ZSMDB079-16	-----	-----	ZSMDB079-16	ZSMDB079-16	-----	-----
Colymbetes paykulli Erichson, 1837	MB0218	ZSMDB080-16	ZSMDB080-16	ZSMDB080-16	ZSMDB080-16	ZSMDB080-16	ZSMDB080-16	ZSMDB080-16	ZSMDB080-16	ZSMDB080-16	ZSMDB080-16	-----	ZSMDB080-16	-----
Colymbetes schildknechti Dettner, 1983	IR56	-----	ZSMDB078-16	-----	-----	ZSMDB078-16	-----	ZSMDB078-16	-----	-----	-----	-----	-----	-----
Colymbetes sculptilis Harris, 1829	IR566	ZSMDB081-16	-----	ZSMDB081-16	-----	ZSMDB081-16	ZSMDB081-16	ZSMDB081-16	ZSMDB081-16	ZSMDB081-16	-----	ZSMDB081-16	ZSMDB081-16	-----
Colymbetes striatus (Linnaeus, 1758)	MB172	ZSMDB082-16	-----	-----	-----	-----	-----	-----	-----	-----	-----	-----	-----	-----
Carabdytes upin Balke et al., 1992	MB0306	KJ637964	KJ637891	FN356769	KJ637873	FN263070	KJ637988	FN257269	ZSMDB006-14	KJ638129	KJ638044	KJ638066	FN256329	HF558697
Hoperius planatus Fall, 1927	MB0187	KJ637961	KJ637889	KJ637927	KJ637869	KJ637974	KJ637983	KJ637906	ZSMDB007-14	-----	KJ638041	KJ638063	-----	----
Meladema coriacea Laporte, 1835	JM0016	ZSMDB076-16	ZSMDB076-16	ZSMDB076-16	-----	ZSMDB076-16	ZSMDB076-16	ZSMDB076-16	ZSMDB076-16	-----	-----	-----	-----	-----
Meladema imbricata (Wollaston, 1871)	MB4793	KJ637969	KJ637898	KJ637937	KJ637881	-----	KJ637993	KJ637919	ZSMDB008-14	KJ638135	-----	-----	-----	----
Meladema lanio (Fabricius, 1775)	JM0015	ZSMDB077-16	ZSMDB077-16	ZSMDB077-16	ZSMDB077-16	ZSMDB077-16	-----	ZSMDB077-16	-----	-----	-----	-----	-----	-----
Melanodytes pustulatus (Rossi, 1792)	MB0330	-----	ZSMDB074-16	ZSMDB074-16	ZSMDB074-16	-----	ZSMDB074-16	ZSMDB074-16	ZSMDB074-16	ZSMDB074-16	ZSMDB074-16	ZSMDB074-16	ZSMDB074-16	-----
Neoscutopterus hornii (Crotch, 1873)	MB0285	KJ637959	KJ637886	KJ637925	KJ637866	-----	KJ637981	KJ637903	ZSMDB009-14	KJ638124	KJ638038	-----	KJ638016	KJ637943
Rhantus alutaceus Fauvel, 1883	MB0119	ZSMDB072-16	ZSMDB072-16	ZSMDB072-16	-----	ZSMDB072-16	ZSMDB072-16	ZSMDB072-16	-----	ZSMDB072-16	ZSMDB072-16	ZSMDB072-16	ZSMDB072-16	-----
Rhantus andinus Balke, 1998a	MB3939	KJ637960	KJ637887	-----	KJ637867	-----	KJ637982	KJ637904	ZSMDB010-14	KJ638125	KJ638039	KJ638061	KJ638017	KJ637944
Rhantus anisonychus Crotch, 1873	MB0160	KJ637965	-----	KJ637930	KJ637874	-----	-----	KJ637910	ZSMDB011-14	KJ638130	KJ638045	-----	KJ638021	KJ637948
Rhantus annectens Sharp, 1882	MB0467	FN257617	FN298848	FN356893	KJ637872	FN263072	KJ637987	FN257278	ZSMDB012-14	KJ638128	KJ638043	KJ638065	FN256331	FN256345
Rhantus antarcticus (Germain, 1854)	MB2694	ZSMDB065-16	-----	-----	-----	-----	-----	-----	ZSMDB065-16	-----	-----	-----	-----	-----
Rhantus atricolor (Aubé, 1838)	MB0157	-----	-----	KJ637935	-----	-----	-----	KJ637917	ZSMDB013-14	KJ638134	KJ638052	-----	KJ638026	KJ637954
Rhantus bacchusi Balke, 2001	MB 1316	ZSMDB032-15	ZSMDB032-15	ZSMDB032-15	ZSMDB032-15	-----	ZSMDB032-15	ZSMDB032-15	-----	ZSMDB032-15	-----	ZSMDB032-15	ZSMDB032-15	ZSMDB032-15
Rhantus binotatus (Harris, 1828)	MB0161	KJ637971	KJ637900	KJ637938	-----	----	-----	KJ637921	ZSMDB014-14	KJ638137	KJ638056	KJ638076	KJ638029	KJ637956
Rhantus bistriatus (Bergsträsser, 1778)	MB0183	FN257606	FN298834	FN356762	-----	FN263061	KJ637990	FN257259	ZSMDB015-14	KJ638131	KJ638048	KJ638069	FN256319	-----
Rhantus bohlei Balke et al., 2002	MB0196	-----	-----	KJ637932	-----	-----	-----	KJ637913	ZSMDB016-14	KJ638132	KJ638049	KJ638070	KJ638024	KJ637951
Rhantus bouvieri Régimbart, 1900	NHRS-JLKB000010349	-----	-----	-----	ZSMDB119-16	-----	-----	ZSMDB119-16	ZSMDB119-16	-----	ZSMDB119-16	-----	ZSMDB119-16	-----
Rhantus bula Balke et al., 2007	MB0477	ZSMDB090-16	-----	ZSMDB090-16	ZSMDB090-16	-----	ZSMDB090-16	ZSMDB090-16	-----	-----	-----	ZSMDB090-16	-----	ZSMDB090-16
Rhantus calidus (Fabrcius, 1792)	MB0338	ZSMDB067-16	ZSMDB067-16	ZSMDB067-16	ZSMDB067-16	-----	-----	ZSMDB067-16	ZSMDB067-16	ZSMDB067-16	ZSMDB067-16	ZSMDB067-16	ZSMDB067-16	-----
Rhantus capensis (Aubé, 1838)	MB0162	ZSMDB116-16	ZSMDB116-16	ZSMDB116-16	-----	ZSMDB116-16	ZSMDB116-16	ZSMDB116-16	ZSMDB116-16	-----	-----	-----	ZSMDB116-16	ZSMDB116-16
Rhantus cheesmanae Balke, 1993	MB1384	KJ637963	FN298824	FN356757	KJ637871	FN263051	KJ637985	FN257249	ZSMDB017-14	-----	-----	-----	KJ638019	KJ637947
Rhantus cicurius (Fabricius, 1787)	MB0049	ZSMDB075-16	ZSMDB075-16	ZSMDB075-16	ZSMDB075-16	ZSMDB075-16	-----	ZSMDB075-16	ZSMDB075-16	ZSMDB075-16	ZSMDB075-16	ZSMDB075-16	ZSMDB075-16	-----
Rhantus consimilis Motschulsky, 1859	MB1232	ZSMDB111-16	ZSMDB111-16	ZSMDB111-16	-----	ZSMDB111-16	ZSMDB111-16	ZSMDB111-16	ZSMDB111-16	ZSMDB111-16	ZSMDB111-16	ZSMDB111-16	ZSMDB111-16	ZSMDB111-16
Rhantus consputus (Sturm, 1834)	MB0182	ZSMDB107-16	ZSMDB107-16	ZSMDB107-16	ZSMDB107-16	ZSMDB107-16	ZSMDB107-16	ZSMDB107-16	ZSMDB107-16	-----	ZSMDB107-16	ZSMDB107-16	ZSMDB107-16	-----
Rhantus dani Balke, 2001	MB3322	ZSMDB095-16	ZSMDB095-16	-----	ZSMDB095-16	ZSMDB095-16	ZSMDB095-16	ZSMDB095-16	ZSMDB095-16	ZSMDB095-16	-----	-----	-----	ZSMDB095-16
Rhantus debilis Sharp, 1882	MB3427	ZSMDB102-16	ZSMDB102-16	ZSMDB102-16	ZSMDB102-16	ZSMDB102-16	ZSMDB102-16	ZSMDB102-16	ZSMDB102-16	-----	ZSMDB102-16	ZSMDB102-16	ZSMDB102-16	ZSMDB102-16
Rhantus ekari Balke & Hendrich, 1992	MB3258	-----	ZSMDB097-16	-----	-----	ZSMDB097-16	ZSMDB097-16	ZSMDB097-16	-----	ZSMDB097-16	ZSMDB097-16	ZSMDB097-16	ZSMDB097-16	ZSMDB097-16
Rhantus elegans C.O. Waterhouse, 1895	MB0072	ZSMDB115-16	ZSMDB115-16	ZSMDB115-16	ZSMDB115-16	ZSMDB115-16	-----	ZSMDB115-16	ZSMDB115-16	-----			ZSMDB115-16	-----
Rhantus elisabethae Balke et al., 2007	MB 1308	ZSMDB034-15	ZSMDB034-15	ZSMDB034-15	ZSMDB034-15	ZSMDB034-15	ZSMDB034-15	ZSMDB034-15	ZSMDB034-15	-----	ZSMDB034-15	ZSMDB034-15	ZSMDB034-15	-----
Rhantus englundi Balke & Ramsdale, 2006	MB1264	ZSMDB101-16	ZSMDB101-16	ZSMDB101-16	ZSMDB101-16	-----	ZSMDB101-16	ZSMDB101-16	ZSMDB101-16	-----	ZSMDB101-16	ZSMDB101-16	ZSMDB101-16	ZSMDB101-16
Rhantus erraticus Sharp, 1884	MB1393	ZSMDB087-16	-----	ZSMDB087-16	ZSMDB087-16	ZSMDB087-16	ZSMDB087-16	ZSMDB087-16	-----	ZSMDB087-16	ZSMDB087-16	ZSMDB087-16	-----	ZSMDB087-16
Rhantus exsoletus (Forster, 1771)	MB0180	KJ637972	FN298831	FN356886	KJ637883	FN263058	KJ637996	FN257256	ZSMDB018-14	KJ638138	KJ638058	KJ638079	FN256316	KJ637957
Rhantus fennicus Huldén, 1982	BMNH 827013	ZSMDB105-16	ZSMDB105-16	ZSMDB105-16	-----	ZSMDB105-16	-----	ZSMDB105-16	-----	-----	-----	-----	ZSMDB105-16	-----
Rhantus frontalis (Marsham 1802)	MB0220	ZSMDB113-16	ZSMDB113-16	ZSMDB113-16	-----	-----	-----	ZSMDB113-16	ZSMDB113-16	-----	ZSMDB113-16	ZSMDB113-16	ZSMDB113-16	-----
Rhantus grapii (Gyllenhal, 1808b)	MB0193	KJ637968	KJ637897	KJ637936	KJ637880	KJ637979	KJ637992	KJ637918	ZSMDB019-14	----	KJ638054	KJ638074	KJ638027	KJ637955
Rhantus guadalcanalensis Balke, 1998	MB2894	-----	ZSMDB071-16	ZSMDB071-16	ZSMDB071-16	ZSMDB071-16	ZSMDB071-16	-----	-----	-----	ZSMDB071-16	ZSMDB071-16	-----	
Rhantus gutticollis (Say, 1830)	MB0198	KJ637958	KJ637884	KJ637923	KJ637864	KJ637973	KJ637980	KJ637901	ZSMDB020-14	KJ638122	KJ638036	KJ638059	KJ638015	KJ637941
Rhantus hispanicus Sharp, 1882	MB1231	ZSMDB108-16	ZSMDB108-16	ZSMDB108-16	ZSMDB108-16	ZSMDB108-16	ZSMDB108-16	ZSMDB108-16	ZSMDB108-16	-----	-----	ZSMDB108-16	-----	-----
Rhantus includens (Walker, 1871)	MB3412	ZSMDB117-16	ZSMDB117-16	-----	ZSMDB117-16	ZSMDB117-16	ZSMDB117-16	ZSMDB117-16	-----	-----	ZSMDB117-16	-----	ZSMDB117-16	ZSMDB117-16
Rhantus incognitus Scholz, 1927	MB3918	----	----	----	----	FN826906	KJ637986	KJ637909	ZSMDB021-14	KJ638127	----	KJ638064	KJ638020	----
Rhantus interclusus (Walker, 1858)	JM0018	ZSMDB091-16	ZSMDB091-16	ZSMDB091-16	ZSMDB091-16	-----	ZSMDB091-16	ZSMDB091-16	-----	ZSMDB091-16	ZSMDB091-16	ZSMDB091-16	ZSMDB091-16	ZSMDB091-16
Rhantus intermedius Balke, 1993	MB3426	ZSMDB103-16	-----	ZSMDB103-16	ZSMDB103-16	ZSMDB103-16	ZSMDB103-16	ZSMDB103-16	-----	-----	ZSMDB103-16	ZSMDB103-16	-----	ZSMDB103-16
Rhantus kini Balke et al., 2007	MB0471	ZSMDB089-16	ZSMDB089-16	ZSMDB089-16	ZSMDB089-16	-----	ZSMDB089-16	ZSMDB089-16	-----	ZSMDB089-16	-----	ZSMDB089-16	ZSMDB089-16	ZSMDB089-16
Rhantus latitans, Sharp, 1882	MB0181	-----	ZSMDB106-16	-----	-----	-----	-----	-----	-----	-----	-----	-----	-----	-----
Rhantus latus (Fairmaire, 1869)	RB049	-----	ZSMDB088-16	-----	ZSMDB088-16	-----	-----	ZSMDB088-16	ZSMDB088-16	-----	ZSMDB088-16	ZSMDB088-16	ZSMDB088-16	-----
Rhantus longulus Régimbart, 1895	MB1542	ZSMDB118-16	ZSMDB118-16	-----	ZSMDB118-16	-----	ZSMDB118-16	ZSMDB118-16	ZSMDB118-16	ZSMDB118-16	ZSMDB118-16	-----	ZSMDB118-16	-----
Rhantus monteithi Balke et al., 2007	MB0033	ZSMDB069-16	ZSMDB069-16	ZSMDB069-16	ZSMDB069-16	-----	ZSMDB069-16	ZSMDB069-16	-----	ZSMDB069-16	ZSMDB069-16	ZSMDB069-16	ZSMDB069-16	-----
Rhantus novaevaledoniae J. Balfour-Browne, 1944	JM0030	ZSMDB140-16	ZSMDB140-16	ZSMDB140-16	ZSMDB140-16	ZSMDB140-16	ZSMDB140-16	ZSMDB140-16	ZSMDB140-16	ZSMDB140-16	-----	ZSMDB140-16	-----	-----
Rhantus orbignyi Balke, 2001	JM004	ZSMDB066-16	-----	-----	ZSMDB066-16	ZSMDB066-16	-----	ZSMDB066-16	-----	ZSMDB066-16	ZSMDB066-16	ZSMDB066-16	ZSMDB066-16	-----
Rhantus ovalis Gschwendtner, 1936	MB 1318	ZSMDB033-15	ZSMDB033-15	-----	ZSMDB033-15	-----	-----	ZSMDB033-15	ZSMDB033-15	ZSMDB033-15	ZSMDB033-15	ZSMDB033-15	ZSMDB033-15	ZSMDB033-15
Rhantus papuanus J. Balfour-Browne, 1939	MB1317	ZSMDB100-16	ZSMDB100-16	ZSMDB100-16	ZSMDB100-16	-----	ZSMDB100-16	ZSMDB100-16	ZSMDB100-16	ZSMDB100-16	-----	-----	ZSMDB100-16	ZSMDB100-16
Rhantus pederzanii Toledo & Mazzoldi, 1996	MB0112	ZSMDB093-16	ZSMDB093-16	ZSMDB093-16	ZSMDB093-16	ZSMDB093-16	ZSMDB093-16	ZSMDB093-16	ZSMDB093-16	ZSMDB093-16	ZSMDB093-16	ZSMDB093-16	-----	ZSMDB093-16
Rhantus phocaenarum Guignot, 1957	MB4790	-----	KJ637888	KJ637926	KJ637868	-----	-----	KJ637905	-----	ZSMDB024-14	KJ638040	KJ638062	-----	KJ637945
Rhantus poellerbauerae Balke et al., 2007	MB0031	ZSMDB070-16	ZSMDB070-16	ZSMDB070-16	ZSMDB070-16	-----	ZSMDB070-16	ZSMDB070-16	ZSMDB070-16	-----	ZSMDB070-16	ZSMDB070-16	ZSMDB070-16	-----
Rhantus pseudopacificus Balke, 1993	MB0150	ZSMDB073-16	ZSMDB073-16	ZSMDB073-16	ZSMDB073-16	ZSMDB073-16	ZSMDB073-16	ZSMDB073-16	-----	ZSMDB073-16	ZSMDB073-16	ZSMDB073-16	-----	-----
Rhantus riedeli Balke, 2001	MB1284	ZSMDB098-16	-----	ZSMDB098-16	ZSMDB098-16	ZSMDB098-16	ZSMDB098-16	ZSMDB098-16	ZSMDB098-16	ZSMDB098-16	-----	ZSMDB098-16	-----	ZSMDB098-16
Rhantus schereri Balke, 1990	MB3424	ZSMDB104-16	ZSMDB104-16	ZSMDB104-16	ZSMDB104-16	ZSMDB104-16	ZSMDB104-16	ZSMDB104-16	ZSMDB104-16	-----	-----	-----	-----	ZSMDB104-16
Rhantus selkirki (Morinière et al., 2014)	MB3836	-----	-----	KJ637939	-----	-----	-----	KJ637922	KJ638014	ZSMDB031-14	-----	KJ638077	-----	-----
Rhantus sericans Sharp, 1882	MB0337	ZSMDB112-16	ZSMDB112-16	ZSMDB112-16	-----	ZSMDB112-16	ZSMDB112-16	ZSMDB112-16	ZSMDB112-16	-----	ZSMDB112-16	ZSMDB112-16	ZSMDB112-16	ZSMDB112-16
Rhantus sexualis Zimmermann, 1919	MB3065	ZSMDB086-16	ZSMDB086-16	-----	ZSMDB086-16	ZSMDB086-16	ZSMDB086-16	ZSMDB086-16	ZSMDB086-16	-----	-----	-----	------	-----
Rhantus signatus (Fabricius, 1775)	MB0159	-----	-----	KJ637929	-----	-----	-----	KJ637908	KJ638003	ZSMDB026-14	-----	-----	-----	-----
Rhantus signatus kuscheli Guignot, 1952	MB4327	-----	KJ637895	KJ637934	KJ637878	-----	-----	KJ637915	-----	ZSMDB022-14	KJ638050	KJ638071	-----	KJ637952
Rhantus signatus spec. (unpublished)	MB1222	-----	KJ637896	-----	-----	-----	-----	KJ637916	KJ638009	ZSMDB025-14	KJ638051	KJ638072	KJ638025	KJ637953
Rhantus sikkimensis Régimbart, 1899	MB0303	ZSMDB092-16	ZSMDB092-16	ZSMDB092-16	-----	ZSMDB092-16	ZSMDB092-16	ZSMDB092-16	-----	ZSMDB092-16	-----	ZSMDB092-16	-----	ZSMDB092-16
Rhantus simulans Régimbart, 1908	MB0284	ZSMDB099-16	ZSMDB099-16	ZSMDB099-16	-----	ZSMDB099-16	ZSMDB099-16	ZSMDB099-16	ZSMDB099-16	ZSMDB099-16	ZSMDB099-16	ZSMDB099-16	ZSMDB099-16	ZSMDB099-16
Rhantus sinuatus (LeConte, 1862)	JM0017	-----	ZSMDB085-16	-----	-----	-----	-----	ZSMDB085-16	-----	-----	-----	-----	ZSMDB085-16	-----
Rhantus socialis (C.O. Waterhouse, 1867)	MB1283	ZSMDB120-16	ZSMDB120-16	ZSMDB120-16	ZSMDB120-16	ZSMDB120-16	ZSMDB120-16	ZSMDB120-16	-----	ZSMDB120-16	ZSMDB120-16	ZSMDB120-16	-----	ZSMDB120-16
Rhantus souzannae Balke, 1990	MB0200	KJ637962	KJ637890	KJ637928	KJ637870	KJ637975	KJ637984	KJ637907	KJ638002	ZSMDB027-14	KJ638042	-----	KJ638018	KJ637946
Rhantus supranubicus Balke, 2001	MB0070	ZSMDB096-16	ZSMDB096-16	ZSMDB096-16	ZSMDB096-16	ZSMDB096-16	ZSMDB096-16	ZSMDB096-16	ZSMDB096-16	-----	ZSMDB096-16	ZSMDB096-16	ZSMDB096-16	ZSMDB096-16
Rhantus suturalis (MacLeay, 1825) (**PAL**)	JM0022	ZSMDB139-16	-----	ZSMDB139-16	ZSMDB139-16	ZSMDB139-16	ZSMDB139-16	ZSMDB139-16	-----	-----	-----	-----	ZSMDB139-16	-----
Rhantus suturalis (MacLeay, 1825) (**AUS**)	MB0006	FN257581	FN298810	FN356775	KJ637882	----	KJ637995	FN257231	FN257532	ZSMDB028-14	KJ638057	KJ638078	FN256293	FN256352
Rhantus suturellus (Harris, 1828)	MB0291	ZSMDB110-16	ZSMDB110-16	ZSMDB110-16	ZSMDB110-16	ZSMDB110-16	ZSMDB110-16	ZSMDB110-16	ZSMDB110-16	-----	ZSMDB110-16	ZSMDB110-16	ZSMDB110-16	ZSMDB110-16
Rhantus tristanicola (Morinière et al.., 2014)	MB3836	-----	-----	KJ637939	-----	-----	-----	KJ637922	KJ638014	ZSMDB031-14	-----	KJ638077	-----	-----
Rhantus validus (Sharp, 1882)	JM0006	ZSMDB068-16	ZSMDB068-16	ZSMDB068-16	ZSMDB068-16	ZSMDB068-16	ZSMDB068-16	ZSMDB068-16	-----	-----	ZSMDB068-16	ZSMDB068-16	ZSMDB068-16	-----
Rhantus vermiculatus Motschulsky, 1860	MB1329	ZSMDB114-16	ZSMDB114-16	-----	-----	-----	-----	ZSMDB114-16	ZSMDB114-16	-----	ZSMDB114-16	ZSMDB114-16	ZSMDB114-16	ZSMDB114-16
Rhantus vicinus (Aubé, 1838)	MB3940	KJ637967	KJ637893	-----	KJ637876	KJ637977	-----	KJ637912	-----	ZSMDB029-14	KJ638047	KJ638068	KJ638023	KJ637950
Rhantus vinsoni Balke, 1992	MB1541	ZSMDB121-16	ZSMDB121-16	-----	ZSMDB121-16	-----	ZSMDB121-16	ZSMDB121-16	-----	ZSMDB121-16	ZSMDB121-16	ZSMDB121-16	-----	ZSMDB121-16
Rhantus vitiensis J.Balfour-Browne, 1944	MB0469	FN257577	FN298849	FN356894	KJ637879	FN263073	KJ637991	FN257279	FN257548	ZSMDB030-14	KJ638053	KJ638073	FN256332	FN257220
Rhantus wallisi Hatch, 1953	MB0289	ZSMDB109-16	ZSMDB109-16	ZSMDB109-16	ZSMDB109-16	-----	-----	ZSMDB109-16	ZSMDB109-16	ZSMDB109-16	ZSMDB109-16	ZSMDB109-16	ZSMDB109-16	ZSMDB109-16

Table S2

Partitioning scheme (BIC) performed in this study.

Subset	Best Model	Subset Partitions
**1**	GTR+I+G	co1a, co1ba, co2a, coba
**2**	GTR+I+G	co1b, co1bb, co2b, cobb
**3**	GTR+G	co1c
**4**	GTR+I+G	arka, cada, enoa, wnga
**5**	SYM+I+G	18s, cadb, enob
**6**	SYM+G	enoc
**7**	GTR+I+G	12s, 16s
**8**	HKY+G	co1bc, co2c, cobc
**9**	JC+I+G	arkb, ef1aa, wngb
**10**	JC	ef1ab, h4a, h4b
**11**	K80+G	ef1ac
**12**	SYM+I+G	arkc, wngc
**13**	HKY+I+G	cadc
**14**	GTR+G	h4c

Table S3

Likelihood models obtained by PartitionFinder , with Bayes factor (B_F) estimates, Bayesian inference (BI) harmonic means and *effective sample size* (ESS) values.

Model	BHM	ESS	BIC	AIC	AICc
BIC	-92555.40	5704.3457	-	0	0
AIC	-92306.18	4964.5752	∞	-	∞
AICc	-92516.52	5554.1293	∞	∞	-

Table S4

Results of AUC value calculations for each species.

Species	AUC value
C. crotchi	0.69
C. fuscus	0.76
C. koenigi	0.64
C. paykulli	0.75
C. schildknechti	0.59
C. striatus	0.83
Hop. planatus	0.59
M. coriacea	0.83
M. imbricata	0.83
M. lanio	0.85
Me. pustulatus	0.75
R. alutaceus	0.75
R. andinus	0.75
R. anisonychus	0.7
R. anisonychus	0.73
R. annectens	0.75
R. atricolor	0.69
R. bacchusi	0.8
R. bidistigma	0.65
R. binotatus	0.61
R. bistriatus	0.81
R. bohlei	0.74
R. bouvieri	0.7
R. bula	0.75
R. calidus	0.71
R. capensis	0.60
R. cheesmanae	0.8
R. cicurius	0.65
R. consimilis	0.78
R. consputus	0.86
R. dani	0.8
R. debilis	0.8
R. ekari	0.8
R. elegans	0.53
R. elisabethae	0.65
R. englundi	0.75
R. erraticus	0.75
R. exsoletus	0.74
R. frontalis	0.67
R. grapii	0.63
R. gutticollis	0.69
R. hispanicus	0.77

R. includens	0.65
R. incognitus	0.78
R. interclusus	0.70
R. intermedius	0.8
R. kini	0.8
R. kuscheli	0.9
R. latitans	0.76
R. latus	0.74
R. longulus	0.69
R. monteithi	0.7
R. notaticollis	0.67
R. novocaledoniae	0.75
R. ovalis	0.75
R. papuanus	0.80
R. pederzanii	0.75
R. phoecaenarum	0.95
R. poellerbauerae	0.7
R. pseudopacificus	0.65
R. riedeli	0.7
R. riedeli	0.6
R. schereri	0.75
R. selkirki	0.9
R. sericans	0.63
R. sexualis	0.75
R. signatus	0.71
R. signatus spec	0.71
R. sikkimensis	0.70
R. simulans	0.75
R. sinuatus	0.69
R. socialis	0.7
R. socialis	0.75
R. souzannae	0.64
R. supranubicus	0.9
R. sutuczech	0.71
R. suturalis	0.71
R. suturellus	0.69
R. tristanicola	0.85
R. validus	0.69
R. vermiculatus	0.59
R. vicinus	0.64
R. vinsoni	0.75
R. vitiensis	0.75
R. wallisi	0.62

Table S5

Comparison of YULE vs BIRTH DEATH (Uncorrelated Lognormal and Exponential).

	ESS – effective sample size	Ln P	YULE Unc	YULE Exp	BD Unc	BD Exp
**YULE Unc**	15.1497	-24108.128	-	0	0	0
**YULE Exp**	38.0712	-24023.037	∞	-	0	∞
**BD Unc**	75.5664	-23963.651	∞	∞	-	∞
**BD Exp**	15.3648	-23987.817	∞	∞	0	-

Comparison of uniform vs normal prior distribution for a Birth Death model (Uncorrelated Lognormal)

	ESS – effective sample size	Ln P	Normal	Uniform
**BD Unc Normal**	158.094	-80932.55	-	∞
**BD Unc Uniform**	200.47	-80958.629	∞	-

Table S6 Results from the *TreePar* analyses conducted on the BEAST chronogram.

Pm	-logL	*P*	r1	τ1	st1	r2	τ2	st2	r3	τ3	st3	r4	τ4	st4	r5	τ 5
**2**	**293.243**	**-**	**0.060**	**0.000**	**-**	**-**	**-**	**-**	**-**	**-**	**-**	**-**	**-**	**-**	**-**	**-**
5	290.790	0.179	0.042	0.352	31.800	0.06	0.610	-	-	-	-	-	-	-	-	-
8	287.476	0.073	0.050	0.203	28.800	0.000	0.511	31.800	0.048	0.703	-	-	-	-	-	-
11	285.920	0.101	0.059	0.000	28.800	0.000	0.556	31.800	0.700	0.745	32.800	0.052	0.504	-	-	-
14	284.547	0.135	0.058	0.000	28.800	0.001	0.297	31.800	0.025	0.909	32.800	0.000	0.735	33.600	0.064	0.438
17	283.198	0.169	0.055	0.088	28.800	0.000	0.814	31.800	0.025	0.915	32.800	0.000	0.386	33.600	0.200	0.767

Notes: Pm, number of parameters in the model; -LogL, the log-likelihood of the model; *P*, p-value of the Likelihood Ratio Test between the incrementally more complex models (if *P*<0.05 the model is supported); r1, diversification rate at present; τ1, turnover rate at present; st1, most recent shift time.

Other diversification and turnover rates, as well as shift times, going deeper in the past are denoted with numbers (e.g. r2, τ2, and st2). In bold is underlined the best-fit model

Table S7 – Results of the GeoSSE analysis.

**model**	**lnLik**	**AIC**	**AIC_weights**
4parameters	-423.05	854.11	0.000101946
6parameters	-412.18	836.36	0.729013594
7parameters	-412.17	838.34	0.270884459

Table S8

Results of Blomberg K value calculations.

	**K-value**	**P-value**
**BIO1**	0.332	0.001
**BIO2**	0.362	0.001
**BIO3**	0.309	0.004
**BIO4**	0.353	0.002
**BIO12**	0.341	0.003
**BIO15**	0.310	0.01

Table S9

dAICc statistics for models of trait evolution.

		**BM**	**OU**	**WN**
**BIO1**	AICc	1070.77	1045.78	1060.82
	AICc_SE	1072.89	1047.97	1062.96
	dAICc	24.99	0	15.04
	dAICc_SE	24.92	0	14.99
**BIO2**	AICc	861.90	832.15	846.52
	AICc_SE	863.75	834.34	848.67
	dAICc	29.75	0	14.37

	dAICc_SE	29.41	0	14.32
**BIO3**	AICc	817.58	789.65	800.38
	AICc_SE	819.43	791.84	802.52
	dAICc	27.93	0	10.73
	dAICc_SE	27.59	0	10.68
**BIO4**	AICc	1748.18	1722.12	1734.73
	AICc_SE	1750.32	1724.31	1736.88
	dAICc	26.06	0	12.61
	dAICc_SE	26.01	0	12.56
**BIO12**	AICc	1533.36	1504.57	1519.47
	AICc_SE	1535.50	1506.76	1521.61
	dAICc	28.80	0	14.90
	dAICc_SE	28.75	0	14.85
**BIO15**	AICc	867.79	836.54	838.18
	AICc_SE	869.20	838.73	840.32
	dAICc	31.25	0	1.64
	dAICc_SE	30.47	0	1.59

Legends of Supplementary Figures in numerical order

Figure S1

Contribution of each climatic variable within studied biogeographical regions in percent (PAC – Pacific region, WPAL – Western Palaearctic, EPAL – Eastern Palaearctic, NEO – Neotropics, AFR – Afrotropics, NEA – Nearctic, AUS – Australis). BIO1, BIO2 and BIO4 contributed the most to the model predictions among the different biogeographic regions.

Figure S2

Topology of the Bayesian inference (BI). Posterior probabilities are indicated at each node.

Figure S3

Results of the BEAST analysis. HPD intervals and median ages are indicated at each node.

Figure S4

Results of the TreePar and BAMM diversification rate analyses.

Figure S5

Results of the GeoSSE analyses. (Left panel) Colymbetinae species richness and their latitudinal diversity gradient. (Right panels) Posterior distributions of speciation, extinction,

dispersal and net diversification rates estimate (temperate regions in blue and tropical regions in red). Speciation and dispersal is higher in the tropics, whereas 95% confidence intervals for extinction and net diversification do overlap.

Figure S6

Results of the Niche overlaps computed using the PNO profiles for each climatic variables, using the summary statistics Schoener's D and Hellinger Distances according to their geographic region and a mean PNO was calculated, which gave an estimate of the niche overlap between regions.

Figure S7

Combined PNO data for each climatic variable using the summary statistics Schoener's D and Hellinger Distances.

Supplemental Material A

Topology of the Bayesian Inference (BI) and Maximum Likelihood (ML) on the left side, and preferred maximum parsimony (MP) topology of the five most parsimonious trees on the right side. Node supports for posterior probabilities (PP), bootstrap (BP) and Jackknife values are indicated on each node. Topological differences are highlighted as light blue lines between the topologies. *Rhantus* species groups are indicated by alphabetical characters. A – *Rhantus calidus* group; B – *Rhantus pacificus* group; C – *Melanodytes* group; D – *Hoperius/Neoscutopterus* group; E – *Meladema* group; F – *Colymbetes* group; G – *Bunites distigma*; H – *Nartus* group; I – *Rhantus suturalis* group; J – *Rhantus exsoletus* group; K – *Rhantus frontalis* group; L – *Rhantus signatus* group; M – *Rhantus bohlei* group

Supplemental Material B

All species of Colymbetinae displayed only by their latitudinal occurrence. Endemic species within the Indo Australian Archipelago, only occurring on highland habitats, are highlighted

in red. Species data was compiled from the world catalogue of Dytiscidae by Nilsson 2013. Map (from Wikipedia) and species richness graphs were created using Microsoft Power Point 2010.

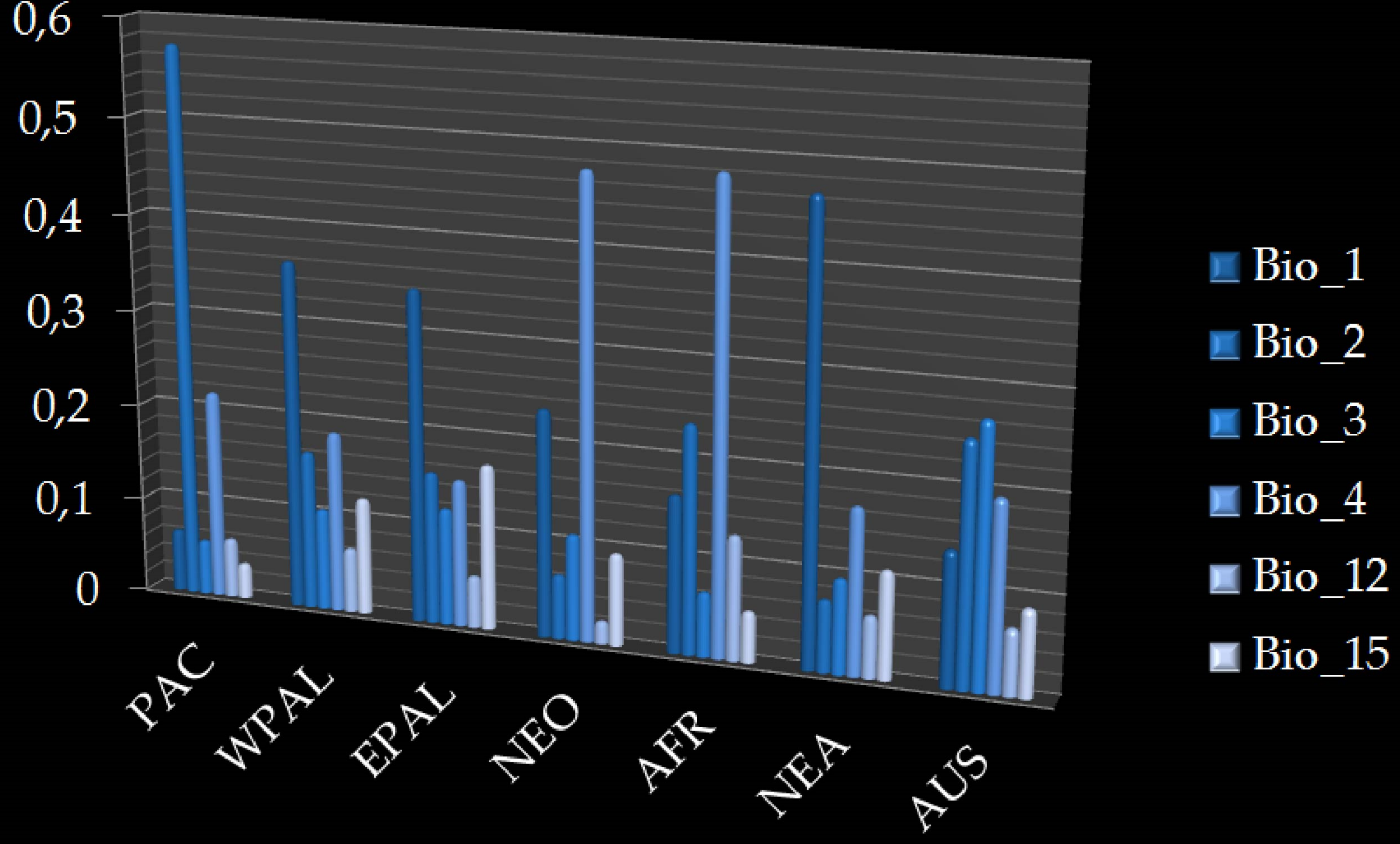

0,6
0,5
0,4
0,3
0,2
0,1
0
PAC
WPAL
EPAL
NEO
AFR
NEA
AUS
Bio_1
Bio_2
Bio_3
Bio_4
Bio_12
Bio_15

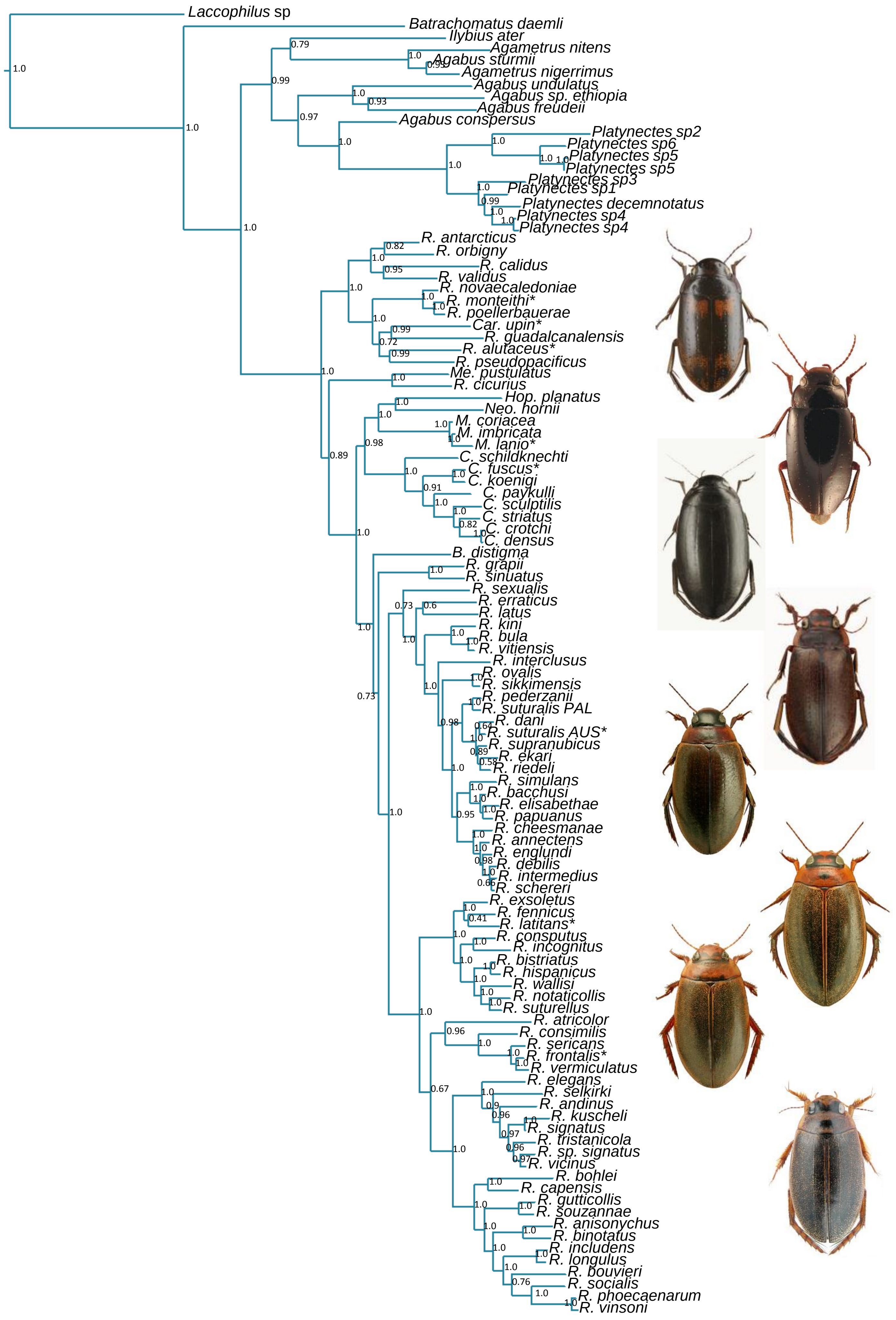

Laccophilus sp
Batrachomatus daemli
Ilybius ater
Agametrus nitens
Agabus sturmii
Agametrus nigerrimus
Agabus undulatus
Agabus sp. ethiopia
Agabus freudeii
Agabus conspersus
Platynectes sp2
Platynectes sp6
Platynectes sp5
Platynectes sp5
Platynectes sp3
Platynectes sp1
Platynectes decemnotatus
Platynectes sp4
Platynectes sp4
R. antarcticus
R. orbigny
R. calidus
R. validus
R. novaecaledoniae
R. monteithi*
R. poellerbauerae
Car. upin*
R. guadalcanalensis
R. alutaceus*
R. pseudopacificus
Me. pustulatus
R. cicurius
Hop. planatus
Neo. hornii
M. coriacea
M. imbricata
M. lanio*
C. schildknechti
C. fuscus*
C. koenigi
C. paykulli
C. sculptilis
C. striatus
C. crotchi
C. densus
B. distigma
R. grapii
R. sinuatus
R. sexualis
R. erraticus
R. latus
R. kini
R. bula
R. vitiensis
R. interclusus
R. ovalis
R. sikkimensis
R. pederzanii
R. suturalis PAL
R. dani
R. suturalis AUS*
R. supranubicus
R. ekari
R. riedeli
R. simulans
R. bacchusi
R. elisabethae
R. papuanus
R. cheesmanae
R. annectens
R. englundi
R. debilis
R. intermedius
R. schereri
R. exsoletus
R. fennicus
R. latitans*
R. consputus
R. incognitus
R. bistriatus
R. hispanicus
R. wallisi
R. notaticollis
R. suturellus
R. atricolor
R. consimilis
R. sericans
R. frontalis*
R. vermiculatus
R. elegans
R. selkirki
R. andinus
R. kuscheli
R. signatus
R. tristanicola
R. sp. signatus
R. vicinus
R. bohlei
R. capensis
R. gutticollis
R. souzannae
R. anisonychus
R. binotatus
R. includens
R. longulus
R. bouvieri
R. socialis
R. phoecaenarum
R. vinsoni

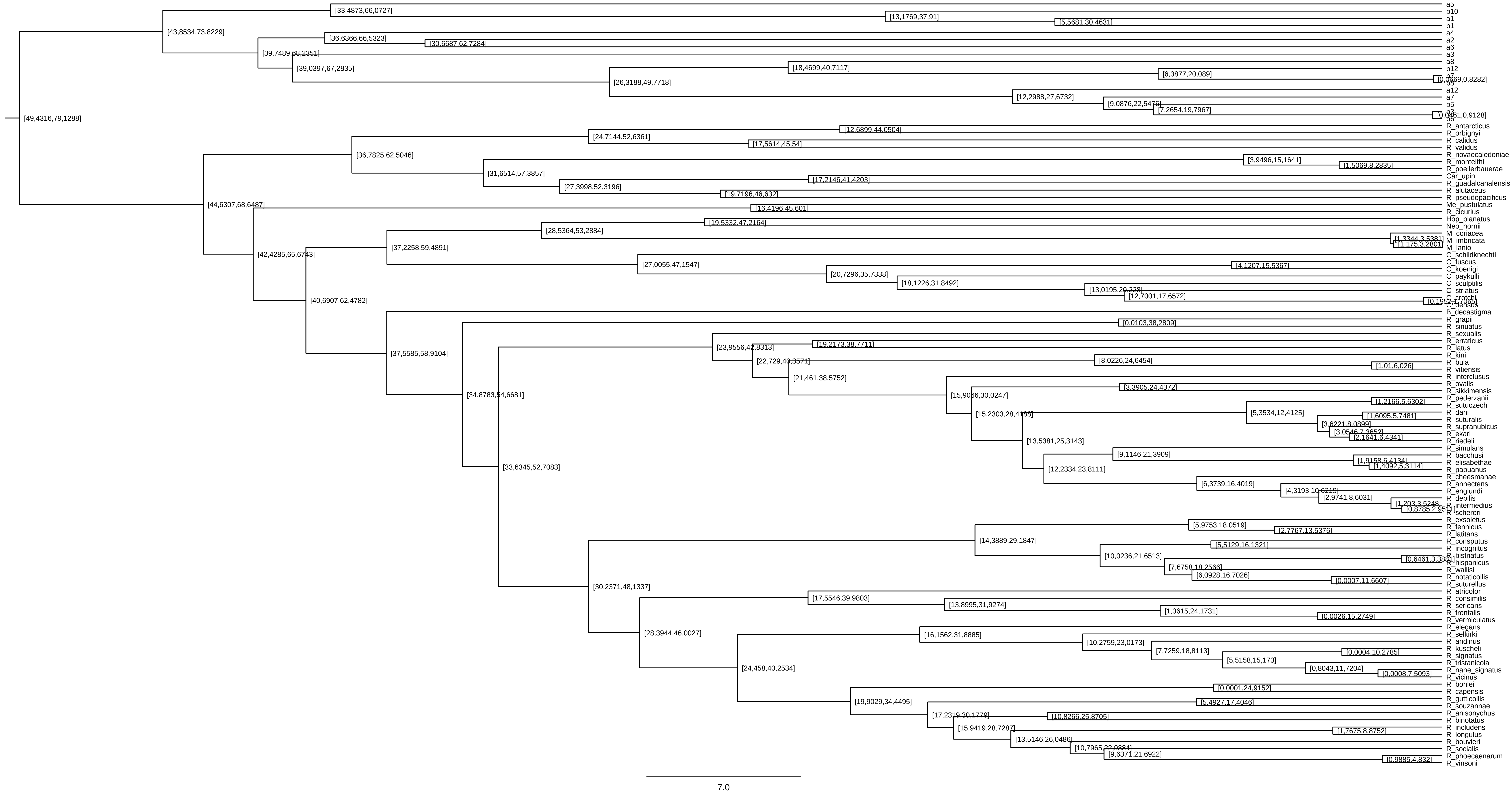
[49,4316,79,1288]
[43,8534,73,8229]
[33,4873,66,0727]
[13,1769,37,91]
[5,5681,30,4631]
[39,7489,68,2351]
[36,6366,66,5323]
[30,6687,62,7284]
[39,0397,67,2835]
[26,3188,49,7718]
[18,4699,40,7117]
[6,3877,20,089]
[0,0669,0,8282]
[12,2988,27,6732]
[9,0876,22,5476]
[7,2654,19,7967]
[0,0461,0,9128]
[44,6307,68,6487]
[36,7825,62,5046]
[24,7144,52,6361]
[12,6899,44,0504]
[17,5614,45,54]
[31,6514,57,3857]
[3,9496,15,1641]
[1,5069,8,2835]
[27,3998,52,3196]
[17,2146,41,4203]
[19,7196,46,632]
[42,4285,65,6743]
[16,4196,45,601]
[40,6907,62,4782]
[37,2258,59,4891]
[28,5364,53,2884]
[19,5332,47,2164]
[1,3344,3,5381]
[1,175,3,2801]
[27,0055,47,1547]
[4,1207,15,5367]
[20,7296,35,7338]
[18,1226,31,8492]
[13,0195,29,228]
[12,7001,17,6572]
[0,1952,1,7065]
[37,5585,58,9104]
[34,8783,54,6681]
[0,0103,38,2809]
[33,6345,52,7083]
[23,9556,42,8313]
[19,2173,38,7711]
[22,729,40,3571]
[8,0226,24,6454]
[1,01,6,026]
[21,461,38,5752]
[15,9066,30,0247]
[3,3905,24,4372]
[15,2303,28,4188]
[1,2166,5,6302]
[5,3534,12,4125]
[1,6095,5,7481]
[3,6221,8,0899]
[3,0546,7,3652]
[2,1641,6,4341]
[13,5381,25,3143]
[9,1146,21,3909]
[1,9158,6,4134]
[1,4092,5,3114]
[12,2334,23,8111]
[6,3739,16,4019]
[4,3193,10,6219]
[2,9741,8,6031]
[1,203,3,5248]
[0,8785,2,9511]
[30,2371,48,1337]
[14,3889,29,1847]
[5,9753,18,0519]
[2,7767,13,5376]
[10,0236,21,6513]
[5,5129,16,1321]
[7,6758,18,2566]
[0,6461,3,3881]
[6,0928,16,7026]
[0,0007,11,6607]
[28,3944,46,0027]
[17,5546,39,9803]
[13,8995,31,9274]
[1,3615,24,1731]
[0,0026,15,2749]
[24,458,40,2534]
[16,1562,31,8885]
[10,2759,23,0173]
[7,7259,18,8113]
[5,5158,15,173]
[0,0004,10,2785]
[0,8043,11,7204]
[0,0008,7,5093]
[19,9029,34,4495]
[0,0001,24,9152]
[17,2319,30,1779]
[5,4927,17,4046]
[15,9419,28,7287]
[10,8266,25,8705]
[13,5146,26,0486]
[1,7675,8,8752]
[10,7965,22,9384]
[9,6371,21,6922]
[0,9885,4,832]
a5
b10
a1
b1
a4
a2
a6
a3
a8
b12
b7
b8
a12
a7
b5
b3
b6
R_antarcticus
R_orbignyi
R_calidus
R_validus
R_novaecaledoniae
R_monteithi
R_poellerbauerae
Car_upin
R_guadalcanalensis
R_alutaceus
R_pseudopacificus
Me_pustulatus
R_cicurius
Hop_planatus
Neo_hornii
M_coriacea
M_imbricata
M_lanio
C_schildknechti
C_fuscus
C_koenigi
C_paykulli
C_sculptilis
C_striatus
C_crotchi
C_densus
B_decastigma
R_grapii
R_sinuatus
R_sexualis
R_erraticus
R_latus
R_kini
R_bula
R_vitiensis
R_interclusus
R_ovalis
R_sikkimensis
R_pederzanii
R_sutuczech
R_dani
R_suturalis
R_supranubicus
R_ekari
R_riedeli
R_simulans
R_bacchusi
R_elisabethae
R_papuanus
R_cheesmanae
R_annectens
R_englundi
R_debilis
R_intermedius
R_schereri
R_exsoletus
R_fennicus
R_latitans
R_consputus
R_incognitus
R_bistriatus
R_hispanicus
R_wallisi
R_notaticollis
R_suturellus
R_atricolor
R_consimilis
R_sericans
R_frontalis
R_vermiculatus
R_elegans
R_selkirki
R_andinus
R_kuscheli
R_signatus
R_tristanicola
R_nahe_signatus
R_vicinus
R_bohlei
R_capensis
R_gutticollis
R_souzannae
R_anisonychus
R_binotatus
R_includens
R_longulus
R_bouvieri
R_socialis
R_phoecaenarum
R_vinsoni
7.0

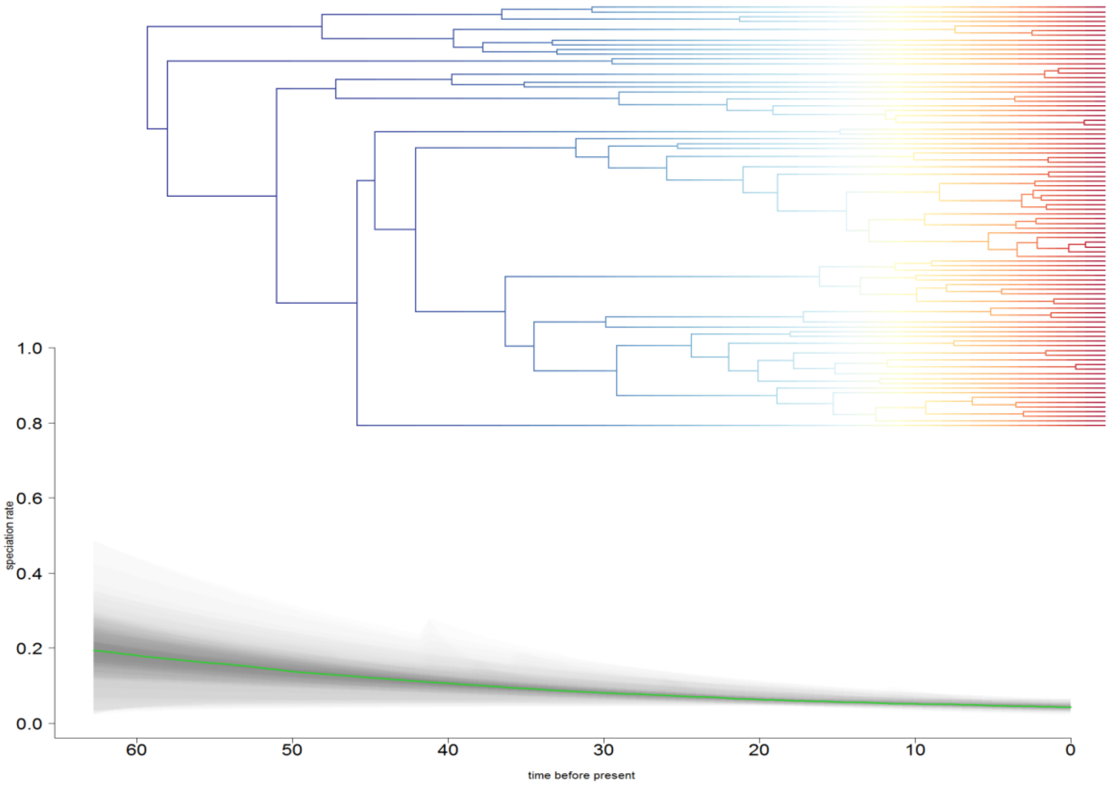
1.0
0.8
0.6
0.4
0.2
0.0
speciation rate
60
50
40
30
20
10
0
time before present

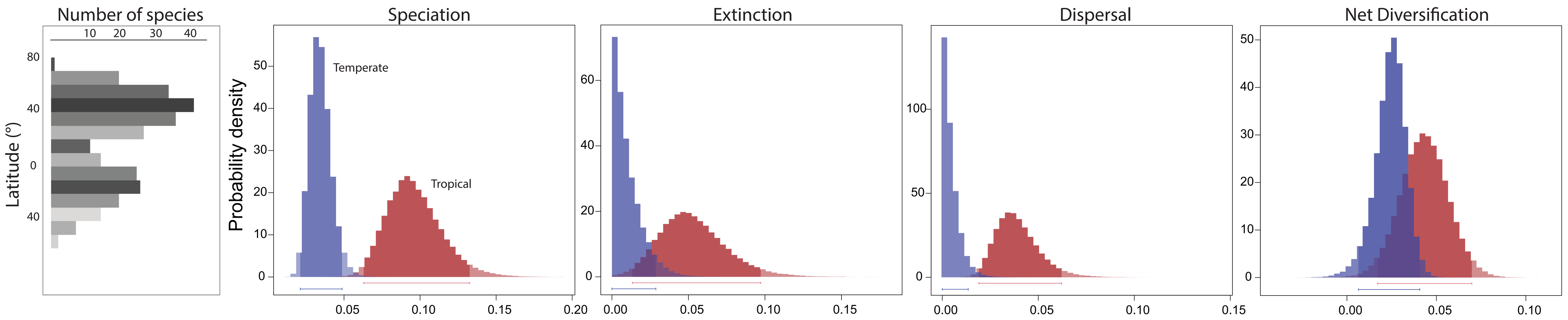

Number of species
10
20
30
40
80
40
0
40
Latitude (°)
Speciation
Probability density
Temperate
Tropical
50
40
30
20
10
0
0.05
0.10
0.15
0.20
Extinction
60
40
20
0
0.00
0.05
0.10
0.15
Dispersal
100
50
0
0.00
0.05
0.10
0.15
Net Diversification
50
40
30
20
10
0
0.00
0.05
0.10

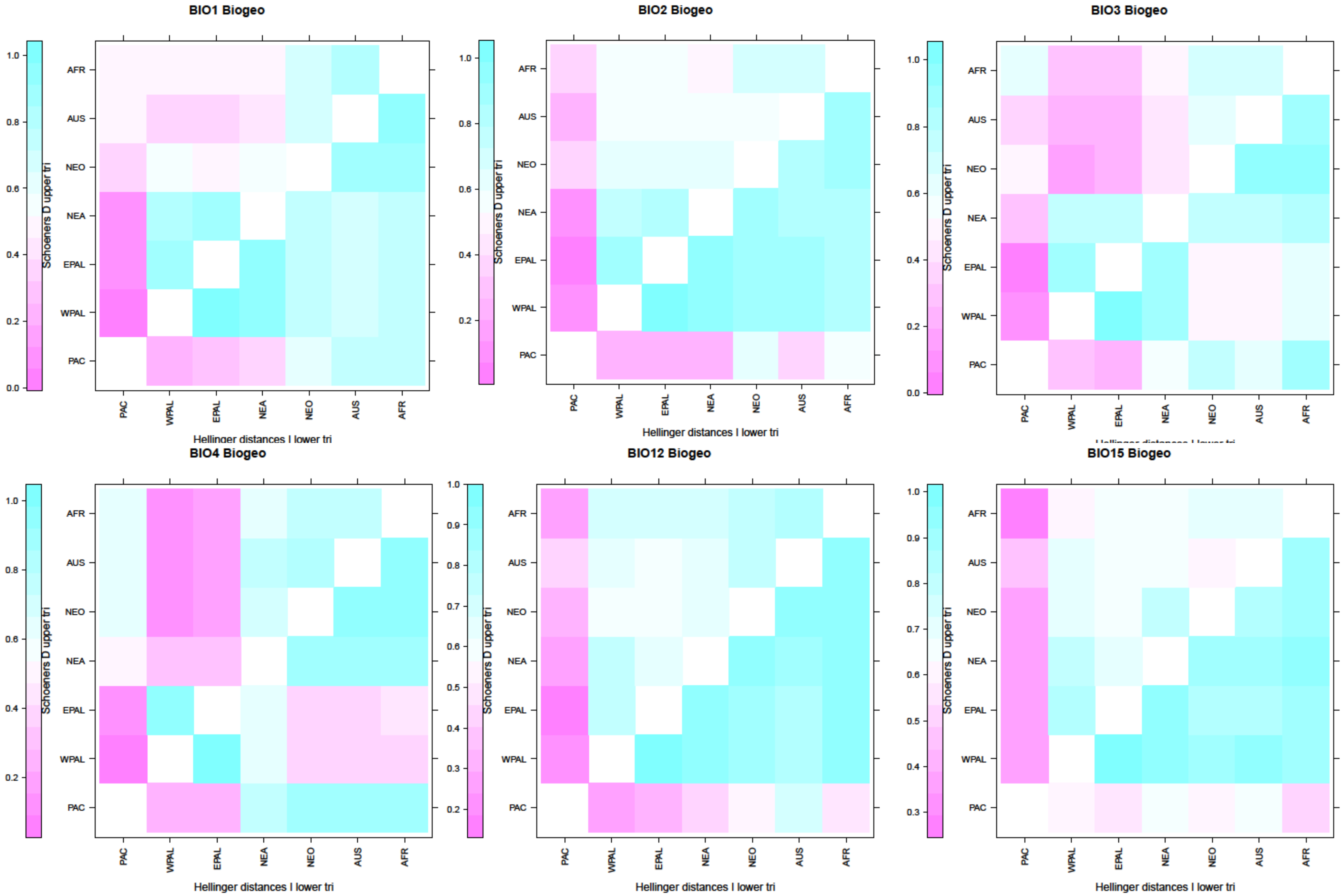
BIO1 Biogeo
BIO2 Biogeo
BIO3 Biogeo
BIO4 Biogeo
BIO12 Biogeo
BIO15 Biogeo
Schoeners D upper tri
Hellinger distances I lower tri
AFR
AUS
NEO
NEA
EPAL
WPAL
PAC

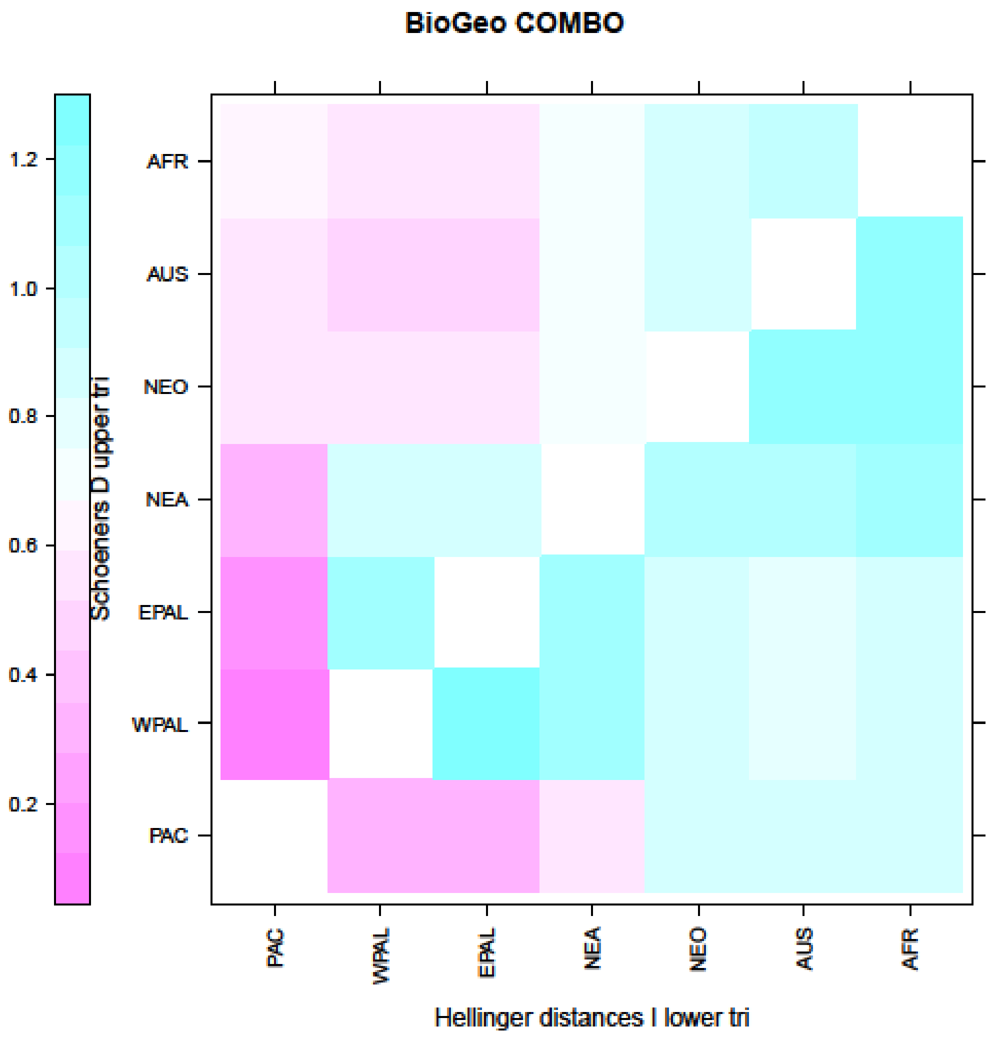
BioGeo COMBO
Schoeners D upper tri
1.2
1.0
0.8
0.6
0.4
0.2
AFR
AUS
NEO
NEA
EPAL
WPAL
PAC
PAC
WPAL
EPAL
NEA
NEO
AUS
AFR
Hellinger distances I lower tri

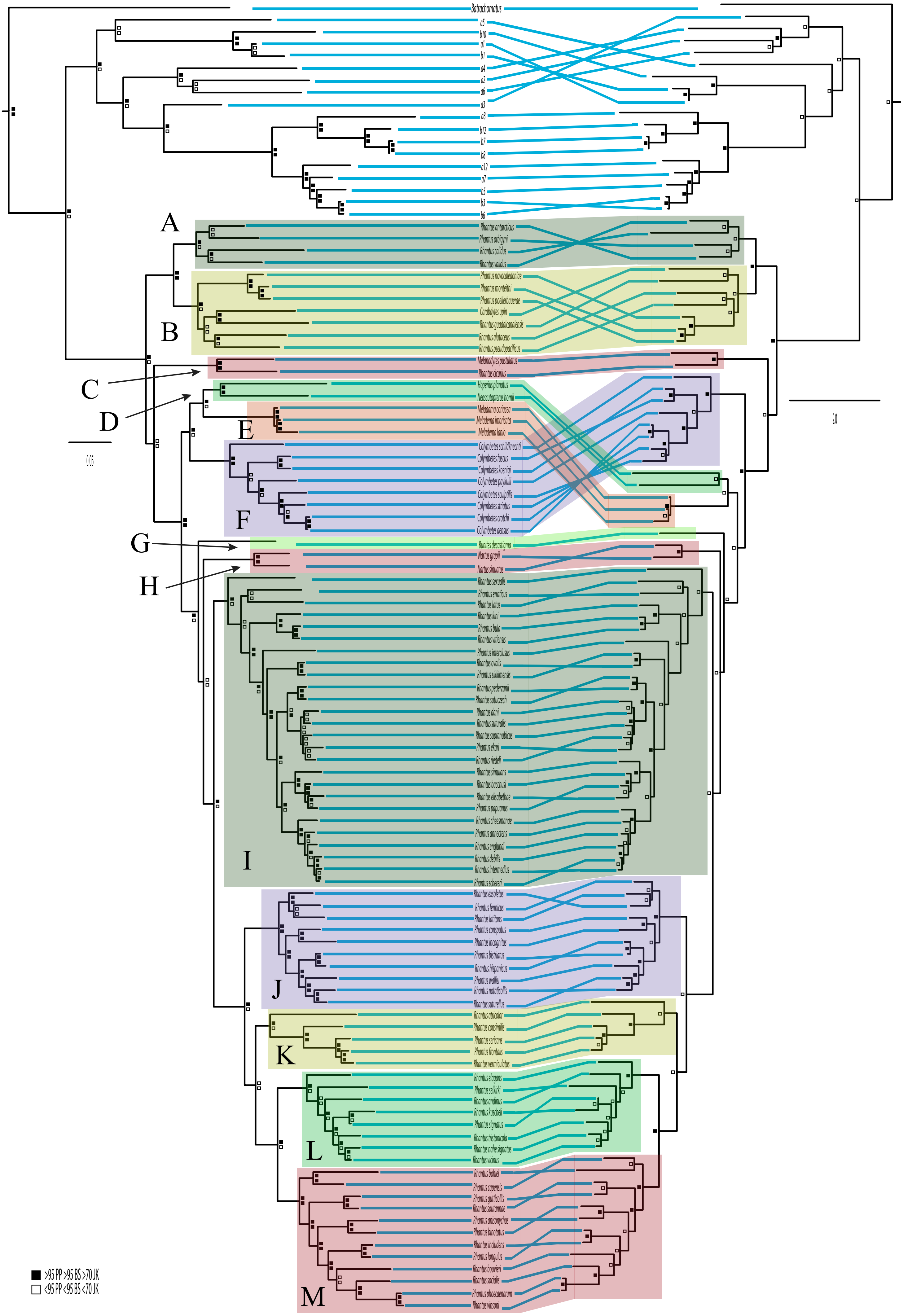
Batrachomatus
a5
b10
a1
b1
a4
a2
a6
a3
a8
b12
b7
b8
a12
a7
b5
b3
b6
A
Rhantus antarcticus
Rhantus orbigyni
Rhantus calidus
Rhantus validus
B
Rhantus novocaledoniae
Rhantus monteithi
Rhantus poellerbauerae
Carabdytes upin
Rhantus guadalcanalensis
Rhantus alutaceus
Rhantus pseudopacificus
C
Melanodytes pustulatus
Rhantus cicurius
D
Hoperius planatus
Neoscutopterus hornii
E
Meladama coriacea
Meladema imbricata
Meladema lanio
F
Colymbetes schildknechti
Colymbetes fuscus
Colymbetes koenigi
Colymbetes paykulli
Colymbetes sculptilis
Colymbetes striatus
Colymbetes crotchi
Colymbetes densus
G
Bunites decastigma
H
Nartus grapii
Nartus sinuatus
I
Rhantus sexualis
Rhantus erraticus
Rhantus latus
Rhantus kini
Rhantus bula
Rhantus vitiensis
Rhantus interclusus
Rhantus ovalis
Rhantus sikkimensis
Rhantus pederzanii
Rhantus sutuczech
Rhantus dani
Rhantus suturalis
Rhantus supranubicus
Rhantus ekari
Rhantus riedeli
Rhantus simulans
Rhantus bacchusi
Rhantus elisabethae
Rhantus papuanus
Rhantus cheesmanae
Rhantus annectens
Rhantus englundi
Rhantus debilis
Rhantus intermedius
Rhantus schereri
J
Rhantus exsoletus
Rhantus fennicus
Rhantus latitans
Rhantus consputus
Rhantus incognitus
Rhantus bistriatus
Rhantus hispanicus
Rhantus wallisi
Rhantus notaticollis
Rhantus suturellus
K
Rhantus atricolor
Rhantus consimilis
Rhantus sericans
Rhantus frontalis
Rhantus vermiculatus
L
Rhantus elagans
Rhantus selkirki
Rhantus andinus
Rhantus kuscheli
Rhantus signatus
Rhantus tristanicola
Rhantus nahe signatus
Rhantus vicinus
M
Rhantus bohlei
Rhantus capensis
Rhantus gutticollis
Rhantus soutannae
Rhantus anisonychus
Rhantus binotatus
Rhantus includens
Rhantus longulus
Rhantus bouvieri
Rhantus socialis
Rhantus phoecaenarum
Rhantus vinsoni
0.05
5.0
>95 PP >95 BS >70 JK
<95 PP <95 BS <70 JK

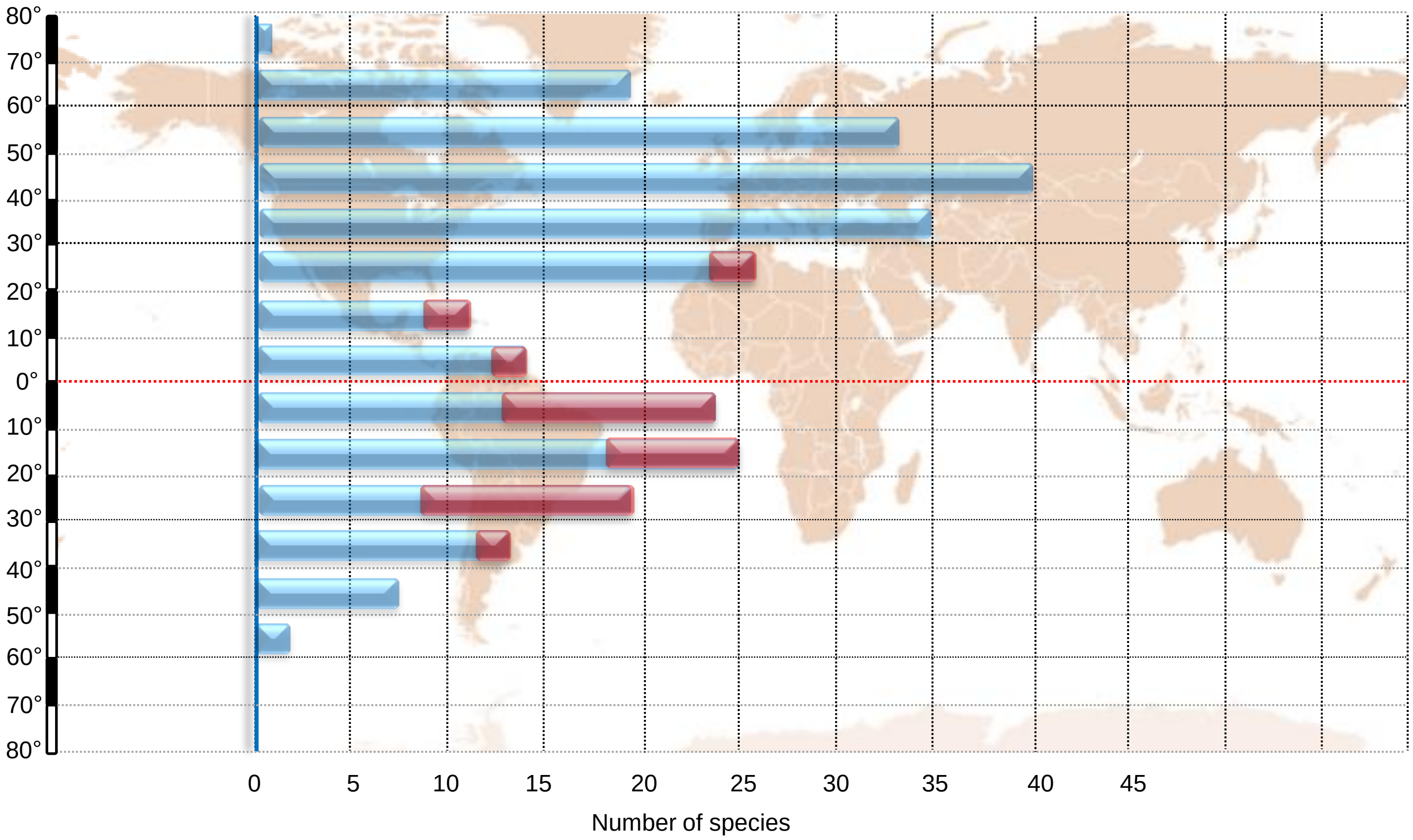
80°
70°
60°
50°
40°
30°
20°
10°
0°
10°
20°
30°
40°
50°
60°
70°
80°
0
5
10
15
20
25
30
35
40
45
Number of species
